# Treatment with EV-miRNAs Alleviates Obesity-Associated Metabolic Dysfunction in Mice

**DOI:** 10.3390/ijms232314920

**Published:** 2022-11-29

**Authors:** Carlos Castaño, Aline Meza-Ramos, Montserrat Batlle, Eduard Guasch, Anna Novials, Marcelina Párrizas

**Affiliations:** 1 Instituto de Investigaciones Biomédicas August Pi i Sunyer (IDIBAPS), 08036 Barcelona, Spain; 2 Centro de Investigación Biomédica en Red de Diabetes y Enfermedades Metabólicas (CIBERDEM), 08036 Barcelona, Spain; 3 Centro de Investigación Biomédica en Red de Enfermedades Cardiovasculares (CIBERCV), 08036 Barcelona, Spain; 4 Cardiovascular Institute, Hospital Clinic, 08036 Barcelona, Spain

**Keywords:** extracellular vesicles, miRNA, HIIT, obesity, cardiometabolic disease, therapy

## Abstract

Most cells release extracellular vesicles (EVs) that can be detected circulating in blood. We and others have shown that the microRNA contents of these vesicles induce transcriptomic changes in acceptor cells, contributing to the adjustment of metabolic homeostasis in response to environmental demands. Here, we explore the potential for modulating obesity- and exercise-derived EV-microRNAs to treat the metabolic dysfunction associated with obesity in mice. Treatment with EV-miRNAs alleviated glucose intolerance and insulin resistance in obese mice to an extent similar to that of high-intensity interval training, although only exercise improved cardiorespiratory fitness and decreased body weight. Mechanistically, EV-miRNAs decreased fatty acid and cholesterol biosynthesis pathways in the liver, reducing hepatic steatosis and increasing insulin sensitivity, resulting in decreased glycemia and triglyceridemia. Our data suggest that manipulation of EV-miRNAs may be a viable strategy to alleviate metabolic dysfunction in obese and diabetic patients who are unable to exercise, although actual physical activity is needed to improve cardiorespiratory fitness.

## 1. Introduction

Obesity is described as excess accumulation of fat mass that can derive from multiple causes. The genetic makeup of the individual, as well as external factors such as perinatal nutrition, diet, lack of physical activity, secondary effects of medical treatments, or even economic factors, can all drive the development of obesity in different individuals [1]. Obesity is not only closely associated with the development of cardiometabolic disorders—such as type 2 diabetes (T2D), metabolic-associated fatty liver disease (MAFLD), or coronary artery disease—but also increases the risk of cancer and worsens the outcomes of concurrent pathologies, as evidenced by the current COVID-19 pandemic [2,3,4]. Inappropriate handling of nutrients by the cells in response to energy demands—termed “metabolic inflexibility”—is one of the underlying causes of obesity-associated hyperglycemia and insulin resistance [5]. A high-calorie diet causes metabolic inflexibility, whereas physical activity favors metabolic flexibility, improves cardiorespiratory fitness (CRF) [5], and delays the onset of T2D, even outperforming the effects of established drugs such as metformin [6]. Indeed, a high CRF predicts lower T2D incidence and greater longevity [7]. However, despite all of its known benefits, adherence to exercise guidelines is low—especially among diabetic and prediabetic subjects [8]. Moreover, for segments of the population such as the elderly, implementation of a complete exercise program might be difficult. Hence, identifying the molecular mechanisms responsible for the beneficial effects of exercise on metabolism could help develop innovative therapies to prevent the metabolic deterioration associated with obesity [9].

In this regard, it is known that exercise facilitates lipid oxidation by the muscles [10] and induces a range of beneficial effects in other tissues, pointing to the stimulation of inter-organ crosstalk. Indeed, during exercise, the muscles secrete a variety of circulating factors, collectively termed “exerkines”, which have been proposed to modulate the function of other tissues in response to exercise [11,12]. Interestingly, exercise also increases the number of small vesicles circulating in the blood [13] and modifies their cargo—particularly their microRNA (miRNA) content [14]. miRNAs are small non-coding RNA molecules that act as post-transcriptional repressors of gene expression [15] and modulate plastic processes—such as metabolic homeostasis—in the face of physiological and pathological stresses [16]. Importantly, aside from their canonical intracellular function, miRNAs can be released by many cells [17], and changes in the profile of extracellular miRNAs are observed in association with diverse pathological conditions—including T2D—providing information regarding their etiology [17,18]. We and others have shown that the miRNAs contained in extracellular vesicles (EV-miRNAs), affect the gene expression of the cells that capture them [19], thereby participating in intercellular communication and regulating insulin sensitivity and the development of metabolic dysfunction [20,21,22,23]. We demonstrated that the injection of control EVs transfected with mimics of the most abundant EV-miRNAs found in the plasma of obese mice—including *miR-122* and *miR-192*—transmitted the phenotype of glucose intolerance, central obesity, and hepatic steatosis to lean mice, suggesting that their inhibition in obese mice may prove to be beneficial [20].

Recently, high-intensity interval training (HIIT)—an exercise modality involving brief bouts of intense activity followed by periods of recovery—has been shown to elicit similar metabolic adaptations to classical endurance exercise training in humans, but with a much shorter time commitment [24,25]. Importantly, HIIT can improve CRF, glycemic control, and insulin sensitivity, all of which are potential risk factors for the development of cardiometabolic disease [26,27,28,29]. We recently characterized the metabolic effects of HIIT in mice and described the EV-miRNA profile established after training. We found that plasma EVs from trained mice were enriched in muscle-specific myomiRs, such as *miR-133b*, while the EV-miRNAs that we previously identified as increased in obesity were significantly decreased. Interestingly, the injection of EVs isolated from the plasma of HIIT mice into sedentary control mice improved glucose tolerance similarly to exercise itself [21].

Indeed, EVs have been shown to have therapeutic applications in various diseases—such as cancer, autoimmune disorders, Alzheimer’s disease, epilepsy, or Parkinson’s disease—by acting as drug delivery carriers [30,31]. Mesenchymal stem cells (MSCs), in particular, have been frequently used as a source of EVs with therapeutic properties, transferring miRNAs to hepatocytes to ameliorate MAFLD in rodent models of obesity [32,33], or to glomerular podocytes to protect them from nephropathy [34]. To improve the efficacy of the EVs, the source cells can be somehow modified—for instance, by subjecting them to hypoxia to increase the release of exosomal miRNAs favoring adaptability to hypoxia, thereby accelerating wound healing [35], or by overexpressing the neuron-specific rabies viral glycoprotein (RVG) peptide, thereby ensuring delivery of the content specifically to neuronal cells [36].

Here, our aim was to test the potential of modulating EV-miRNAs to alleviate the metabolic dysfunction associated with obesity in mice. To this end, we treated obese mice with EVs engineered to carry a miRNA pattern similar to that induced by exercise, with inhibitors of obesity-associated miRNAs and mimics of exercise-induced myomiRs. We studied the metabolic and transcriptomic responses of the treated mice and compared them with the effects of exercise. The primary objective of our study was to obtain an improvement in glucose homeostasis in EV-treated mice similar to that provided by exercise, as measured by a decrease in the glucose area under the curve (AUC) during a glucose tolerance test (GTT).

## 2. Results

### 2.1. Experimental Design, Exogenous EVs’ Biodistribution, and Characterization of Obese Mice

**Figure 1:**
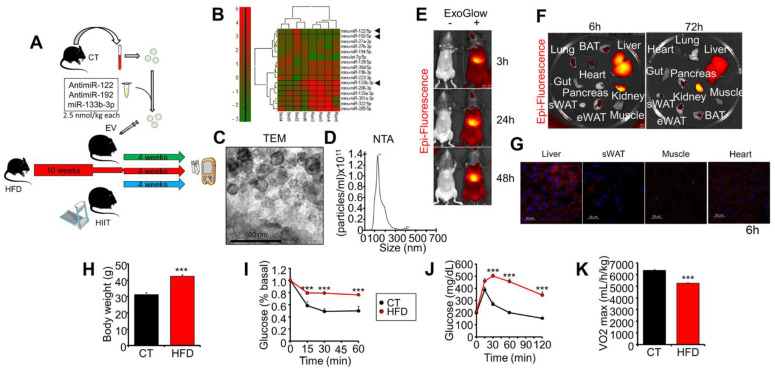
Experimental design, exogenous EVs’ biodistribution, and characterization of obese mice: (**A**) Experimental design. Mice fed with an HFD for 10 weeks were randomly distributed into 3 groups: untreated (HFD, red), exercised (HIIT, blue), and injected with EVs from control mice (CT) transfected with a mix of miRNA mimics and inhibitors (EV, green). All three groups were fed the same high-fat diet during the experimental period. (**B**) Heatmap of exosomal miRNAs isolated from the plasma of sedentary or exercised CT mice, identifying *miR-133b* as one of the miRNAs more upregulated by exercise, whereas *miR-122* and *miR-192* are decreased. Arrowheads indicate selected miRNAs. (**C**,**D**) EVs were characterized by transmission electron microscopy (**C**) and nanoparticle tracking analysis (**D**). (**E**–**G**) Injection of EVs labeled with ExoGlow into CT mice showing rapid accumulation in the liver (**E**,**F**), but a significant signal is also detected in other tissues (**G**). (**H**–**J**) After 10 weeks of high-fat feeding, the mice are obese (**H**), insulin-resistant (**I**), and glucose-intolerant (**J**). (**K**) Obese mice show decreased maximal VO_2_ levels in a capacity test on a treadmill. n = 6 (CT), n = 18 (HFD) (**H**–**K**); *** *p* < 0.005 with respect to the CT group.

We transfected plasma EVs from control mice with a mix of miRNA mimics and inhibitors and used them to treat obese mice (EV) (Figure 1A). We chose to inhibit *miR-122* and *miR-192*—two liver-enriched miRNAs that are increased by obesity and decreased by exercise—and to overexpress muscle-specific *miR-133b*, which is abundant in EVs isolated from the plasma of trained mice (Figure 1B). Another group of obese mice underwent HIIT in parallel (HIIT), whereas a third group was left untreated (HFD). In a preparative experiment, we observed that treatment of both lean and obese mice with native EVs isolated from the plasma of control mice had no effect on their metabolic parameters as compared with non-treated mice (Supplementary Figure S1A).

We isolated EVs from the plasma of control mice by ultracentrifugation and characterized them by transmission electron microscopy (TEM), nanoparticle tracking analysis (NTA), and Western blotting of EV membrane markers CD9 and CD63 (Figure 1C,D and Figure S1B). We studied the biodistribution of exogenous EVs by injecting control mice with EVs fluorescently labeled with ExoGlow (Figure 1E). We observed a rapid accumulation in the liver that was maintained for at least 72 h. However, in the necropsies performed at this time point and at 6 h, we found signals in several other tissues, including the pancreas, kidneys, lungs, heart, and the epididymal, subcutaneous, and brown adipose depots (Figure 1F). At the microscopic level, we observed a high signal in the liver at 6 h and 72 h after injection (Figure 1G and Figure S1C).

After 10 weeks of HFD, the mice were obese (Figure 1H) and replicated different features of prediabetes, including insulin resistance (Figure 1I) and glucose intolerance (Figure 1J and Figure S1D), although their basal glycemia was unaltered (Supplementary Figure S1E). Moreover, HFD mice also showed a lower maximal VO_2_ consumption (VO_2_max) with respect to a group of lean mice fed a standard diet (CT) when subjected to a capacity test on a treadmill (Figure 1K), indicating decreased CRF on top of the metabolic alterations.

### 2.2. HIIT and EV-miRNAs Improve the Metabolic Profile of HFD Mice, but Only HIIT Enhances CRF and Promotes Cardiac Remodeling

**Figure 2:**
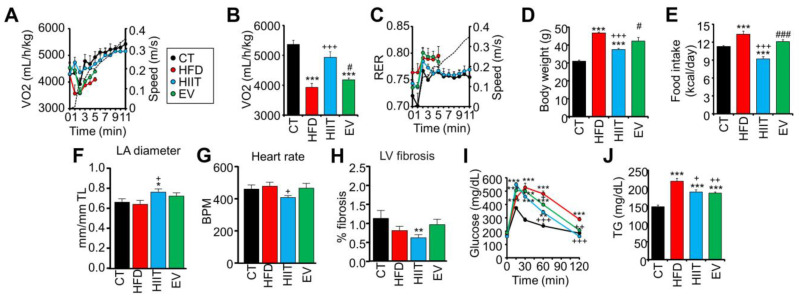
HIIT and EV-miRNAs improve the metabolic profile of HFD mice, but only HIIT enhances CRF and promotes cardiac remodeling: (**A**–**C**) Obese mice trained by HIIT showed increases in their time spent running (**A**), maximal VO_2_ (**B**), and metabolic flexibility (**C**) during a capacity test on a treadmill. Treatment with EV-miRNAs did not affect these parameters. (**D**,**E**) HIIT, but not EV treatment, significantly decreased the body weight of obese mice (**D**), associated with lower food intake (**E**). (**F**–**H**) Trained mice developed significant sinus bradycardia (**F**), left atrial dilation (**G**), and showed decreased myocardial fibrosis in the left ventricle (**H**). (**I**,**J**) Both HIIT and EV improved glucose tolerance (I) and decreased plasma triglyceride levels (**J**). n = 6/group (**A**–**J**); * *p* < 0.05, ** *p* < 0.01, *** *p* < 0.005 with respect to the CT group; + *p* < 0.05, ++ *p* < 0.01, +++ *p* < 0.005 with respect to the HFD group; # *p* < 0.05, ### *p* < 0.005 between the HIIT and EV groups.

To compare the effects of the EV-miRNA therapy with the effects of exercise on CRF parameters, we repeated the capacity test at the end of the 4 weeks of the experimental period. Whereas untreated HFD mice stopped running after 5 min, obese HIIT mice were able to keep running for as long as the lean untrained CT mice (Figure 2A) and reached a comparable level of VO_2_max (Figure 2B). In addition, HFD mice had a high respiratory exchange ratio (RER) throughout the capacity test (Figure 2C), but HIIT mice had decreased RER levels similar to those of CT mice, indicating a greater ability to use lipids as substrates during exercise. Finally, HIIT reduced both body weight and caloric intake with respect to the group of untreated HFD mice (Figure 2D,E). The treatment with EV-miRNAs did not reproduce any of these effects.

On the other hand, HIIT mice also exhibited some symptoms of athlete’s heart, such as left atrial dilation (Figure 2F) and sinus bradycardia (Figure 2G), and showed reduced left ventricular fibrosis compared with CT mice (Figure 2H). EV mice failed to reproduce any of these exercise-induced cardiac remodeling effects. Additionally, other echocardiographic and electrocardiographic parameters were unaffected by diet or treatment (Supplementary Figure S2A–I) [37].

Surprisingly, despite the lack of impact of the EV-miRNA treatment on parameters related to CRF, cardiac remodeling, or body weight, both EV and HIIT mice showed improved glucose tolerance to similar extents (Figure 2I and Figure S2J,K). Improving glucose homeostasis and decreasing the glucose AUC were our primary objectives; hence, these data suggest that the EV treatment was successful. Moreover, both experimental groups also had reduced levels of circulating triglycerides (TGs) (Figure 2J).

Overall, our data show that the treatment with EV-miRNAs has a beneficial metabolic effect comparable to that of training itself in obese mice, although only exercise can improve CRF parameters and promote cardiac remodeling.

### 2.3. HIIT and EV-miRNAs Improve Glucose Tolerance through Different Mechanisms

**Figure 3:**
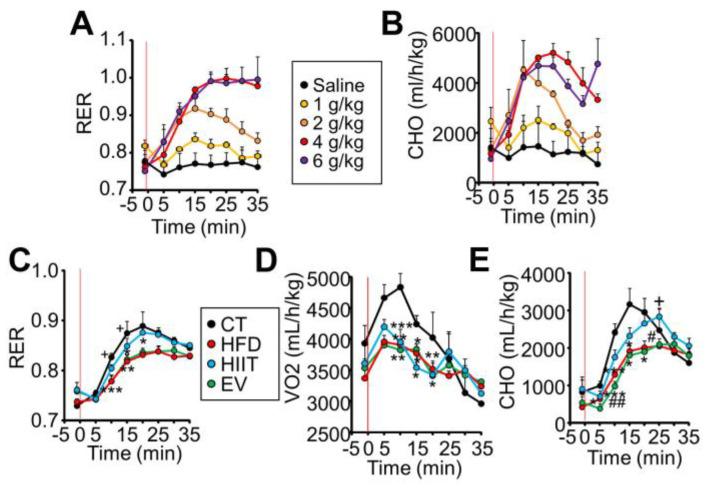
HIIT and EV-miRNAs improve glucose tolerance through different mechanisms: (**A**,**B**) Oral administration of glucose to CT lean mice increased their respiratory exchange ratio (**A**) and carbohydrate oxidation (**B**) in a dose-dependent manner, as measured by calorimetry. (**C**) Untreated HFD mice showed lower RER than CT mice in response to glucose administration, and this was normalized by HIIT but not EVs. (**D**,**E**) Obese mice showed lower O_2_ consumption (**D**) and a partial improvement in carbohydrate oxidation as a result of HIIT (**E**). The red line in the graph indicates the time of glucose administration. N = 2/group (**A**,**B**), n = 5/group (**C**–**E**); * *p* < 0.05, ** *p* < 0.01, *** *p* < 0.005 with respect to the CT group; + *p* < 0.05 with respect to the HFD group; # *p* < 0.05, ## *p* < 0.01 between the HIIT and EV groups.

To study the influence of both treatments on metabolic flexibility, mice were subjected to a GTT while gas exchange was measured by indirect calorimetry on an airtight, stationary, single-lane treadmill. First, to determine the validity of this strategy to assess substrate use in response to intake, we administered increasing oral doses of glucose to control mice and monitored their gas exchange [38]. As expected, we observed that RER increased in response to the increasing glucose doses (Figure 3A and Figure S3A), revealing the ability of control mice to uptake and metabolize glucose, thereby maintaining glycemia within normal limits, even with the saturating 6 g/kg dose (Supplementary Figure S3B). By calculating carbohydrate (CHO) and fat (FAO) oxidation (Figure 3B and Figure S3C) from the O_2_ uptake (VO_2_) and CO_2_ release (VCO_2_) values (Supplementary Figure S3D,E) [39], we observed higher levels of CHO at the expense of lower levels of FAO in the mice administered with increasing doses of glucose, indicating that the control mice were metabolically flexible and could promptly change their substrate use in response to availability.

We next performed the same test with the experimental groups, using a 2 g/kg dose. Untreated HFD mice were unable to increase their RER in response to glucose, pointing to metabolic inflexibility (Figure 3C and Figure S3F). Additionally, HFD mice showed lower VO_2_ than lean CT mice (Figure 3D), resulting in lower overall CHO (Figure 3E), which would explain the hyperglycemia observed during the GTT (Figure 2J). Surprisingly, although HIIT and EV mice showed similar improvements in glucose tolerance (Figure 2J and Figure S2I), calorimetric analysis revealed that only HIIT mice could increase their RER in response to glucose (Figure 3C and Figure S3F). However, despite improved metabolic flexibility, HIIT mice still showed lower VO_2_ and VCO_2_ values than CT mice during the test (Figure 3D and Figure S3G), probably indicating the failure of mitochondria to entirely recover from the metabolic stress associated with obesity [40]. The VO_2_ uptake of HIIT mice was similar to that of untreated HFD mice; therefore, HIIT mice still showed lower CHO levels than CT mice (Figure 3E), which could explain the only partial recovery of glucose tolerance observed (Figure 2J). The increase in CHO in CT and HIIT mice is consistent with them also being the groups with the lowest FAO during the test (Supplementary Figure S3H). EV mice, on the other hand, showed RER and VO_2_ values indistinguishable from those of untreated HFD mice, resulting in similar CHO levels (Figure 3C–E), even though they showed an improvement in glucose tolerance comparable to that of trained mice (Figure 2J).

Overall, even though both treatments improve glucose tolerance to similar extents, the underlying mechanism is different.

### 2.4. Treatment with EV-miRNAs Improves Hepatic Insulin Sensitivity and Steatosis

**Figure 4:**
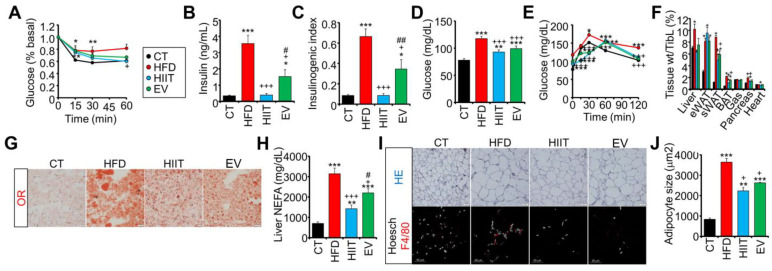
Treatment with EV-miRNAs improves hepatic insulin sensitivity and steatosis: (**A**–**C**) Both HIIT and EV improved the insulin sensitivity of obese mice as measured by an insulin tolerance test (**A**), decreased fasting insulinemia (**B**), and lower insulinogenic index (**C**). (**D**) Both the HIIT and EV groups showed lower glycemia after a long fast than untreated HFD mice. (**E**) Pyruvate tolerance test showed lower gluconeogenesis in HIIT and EV mice as compared to untreated HFD mice. (**F**) Postmortem tissue analysis evidenced decreased liver and sWAT weight in the HIIT and EV groups. (**G**,**H**) Untreated HFD mice showed high levels of hepatic steatosis as determined by Oil Red O staining (**G**) and the quantification of liver non-esterified fatty acids (**H**). Scale bars represent 20 µm. These parameters were partially decreased by both HIIT and EV treatments. (**I**,**J**) HIIT and EV treatments partially reduced adipocyte size as determined by hematoxylin and eosin staining in the sWAT (**I** (upper panels),**J**) and decreased F4/80 macrophage infiltration (**I** (lower panels)). Scale bars represent 50 µm. n = 6/group (**A**–**F**), n = 2/group (**G**–**J**); * *p* < 0.05, ** *p* < 0.01, *** *p* < 0.005 with respect to the CT group; + *p* < 0.05, +++ *p* < 0.005 with respect to the HFD group; # *p* < 0.05, ## *p* < 0.01 between the HIIT and EV groups.

As the improvement of glucose tolerance in EV mice cannot be ascribed to increased glucose utilization, we hypothesized that EV-miRNAs enhanced hepatic insulin sensitivity and gluconeogenesis, thereby maintaining postprandial glycemia by decreasing hepatic glucose output instead of by increasing oxidation. In support of this notion, both HIIT and EV mice showed improved insulin sensitivity (Figure 4A) and reduced basal hyperinsulinemia, highlighting the requirement for lower insulin levels to maintain glycemic control (Figure 4B). Accordingly, both HIIT and EV mice showed a lower insulinogenic index (Figure 4C), which was further associated with reduced pancreatic islet size and total insulin area (Supplementary Figure S4A,B). Moreover, plasma glucose after a prolonged fast was lower in HIIT and EV mice as compared to HFD mice (Figure 4D). Both treatments also showed lower hepatic glucose output than untreated HFD mice during a pyruvate tolerance test (PTT) (Figure 4E).

Interestingly, although only HIIT mice showed lower body weight (Figure 2D), both the HIIT and EV groups showed significant reductions in liver weight at the necropsy (Figure 4F), even though there were no differences in body size as determined by tibia length (Supplementary Figure S4C). Additionally, both treatments decreased hepatic steatosis (Figure 4G) and the contents of non-esterified fatty acids (NEFAs) and TGs in the liver, despite receiving the same high-fat diet as the untreated HFD mice (Figure 4H and Figure S4D). This was associated with decreased abundance of the key lipogenic FASN enzyme in all obese groups, but particularly in EV mice (Supplementary Figure S4E).

Surprisingly, neither intervention reduced the weight of the epididymal adipose tissue (eWAT), although the subcutaneous adipose tissue (sWAT) was significantly reduced by both treatments (Figure 4F). Furthermore, the size of sWAT adipocytes (Figure 4I (upper panels),J) and macrophage infiltration (Figure 4I (lower panels)) were decreased by both interventions. These changes were not observed in the eWAT (Supplementary Figure S4F,G).

Altogether, treatment with EV-miRNAs improves hepatic sensitivity and steatosis, suggesting that this may be their main target tissue.

### 2.5. HIIT and EV-miRNAs Affect the Hepatic, sWAT, and Muscle Expression Profiles of Obese Mice

**Figure 5:**
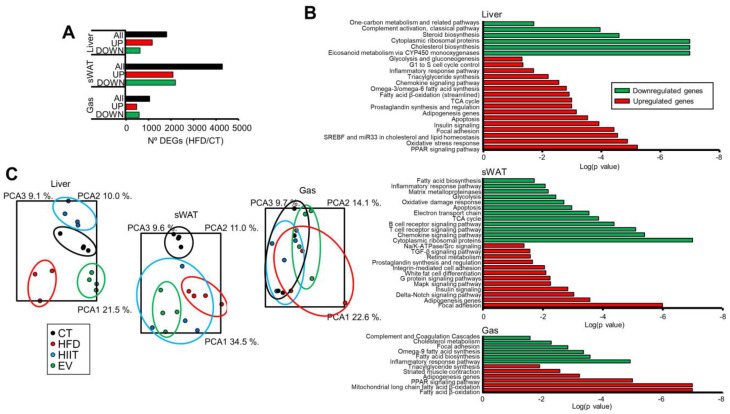
HIIT and EV-miRNAs affect the hepatic, sWAT, and muscle expression profiles of obese mice: (**A**) Number of total (black), upregulated (red), and downregulated (green) genes in the liver, sWAT, and gastrocnemius muscle that were significantly altered in HFD mice at least 1.5-fold in either direction; *p* < 0.05. (**B**) Enrichment analysis showing the log(*p*-value) of pathways associated with upregulated (red) or downregulated genes (green) in the same tissues of HFD mice. (**C**) Principal component analysis in these tissues showing that hepatic gene expression is strongly influenced by diet and treatment. n = 4/group (**A**–**C**).

We analyzed gene expression in the liver, sWAT, and the gastrocnemius muscle by microarray hybridization (n = 4/group). Comparison of the untreated obese HFD mice with the lean CT group identified a high number of differentially expressed genes (DEGs) in all three tissues, with sWAT displaying the most changes and the muscle showing the fewest (Figure 5A and Figure S5A). Enrichment analysis of DEGs identified alterations in many pathways for each tissue (Supplementary Tables S1–S3). To obtain a more detailed picture, we analyzed upregulated and downregulated genes separately (Figure 5B). Upregulated genes in the liver were mainly related to inflammation (*Nfkb1*, *Ccl3*, *Tlr4*) and metabolism (*Slc2a1*, *Gck*, *Apoa4*, *Fabp4*), with decreased cholesterol, steroid, and eicosanoid synthesis (*Elovl3, Hsd3b4, Cyp4a12a*). On the other hand, upregulated genes in sWAT revealed enrichment of pathways related to adipogenesis (*Bmp3*, *Lep*) and cell signaling (*Jun*, *Fos*, *Mras*), whereas the muscle displayed significantly upregulated fatty acid β-oxidation (*Cd36*, *Cpt2*).

We next analyzed the effects of the HIIT and EV treatments on the transcriptomic profiles of obese HFD mice. Again, sWAT was the tissue showing the most changes in gene expression, while the muscle was the least affected by both treatments (Supplementary Figure S5B,C). Principal component analysis (PCA) indicated that hepatic gene expression was strongly influenced by diet and treatment (Figure 5C). Conversely, PCA was unable to distinguish the groups in the muscle. Hierarchical clustering of HFD/CT DEGs showed that the effects of HIIT and EV in sWAT were the most similar. On the other hand, HIIT exerted the greatest effect in the muscle, approximating the gene expression pattern found in the CT group rather than in the HFD mice (Supplementary Figure S5D).

### 2.6. HIIT and EV-miRNAs Partially Reverse the Alterations Induced by Diet

**Figure 6:**
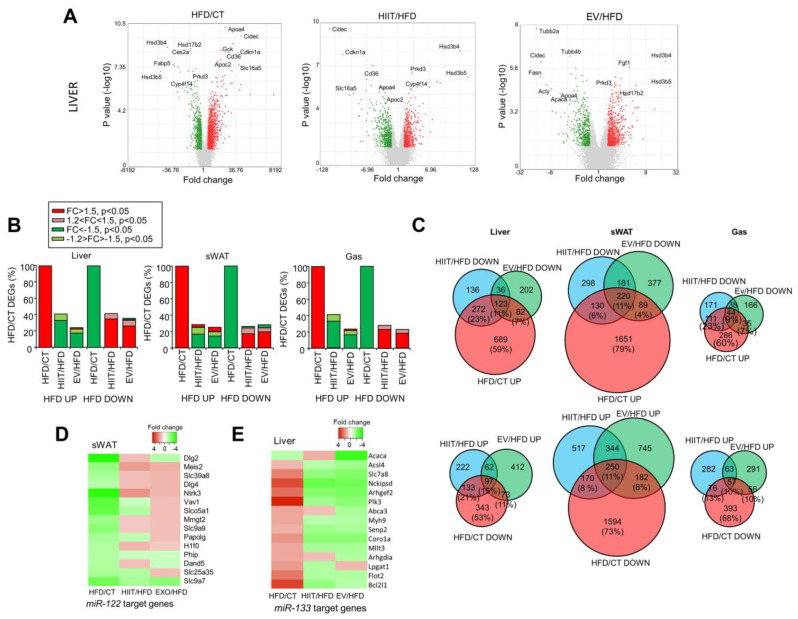
HIIT and EV-miRNAs partially reverse the alterations induced by diet: (**A**) Volcano plots depicting hepatic expression changes induced by the diet (HFD/CT, left panels) or HIIT and EV treatments (HIIT/HFD and EV/HFD; middle and right panels, respectively). (**B**) Histograms showing the percentages of differentially expressed genes in HFD reverted by the HIIT or EV treatments in the liver, sWAT, and gastrocnemius muscle. (**C**) Venn diagrams comparing differentially expressed genes that were upregulated (upper panels) or downregulated (lower panels) in the liver (left panels), sWAT (middle panels), and gastrocnemius muscle (right panels) of HFD mice vs. CT with differentially expressed genes that were downregulated or upregulated (respectively) by each of the treatments as compared with untreated HFD mice. (**D**,**E**) Target genes of *miR-122* were downregulated in obese sWAT and upregulated by EV treatment (**D**), whereas *miR-133* targets were upregulated in the liver and downregulated by EV treatment (**E**). n = 4/group (**A**–**E**).

We speculated that the beneficial metabolic effects of the HIIT and EV treatments might be detected as a total or partial reversion of some of the DEGs observed in obese mice as compared with CT mice. Therefore, we compared the gene expression changes observed in HFD/CT with those induced by each treatment upon HFD expression (Figure 6A and Figure S6A,B). Importantly, the main effects of both the HIIT and EV treatments were to reverse the gene expression changes observed in untreated HFD mice. For instance, hepatic genes downregulated by the diet—such as the enzymes *Hsd3b4* and *Hsd3b5*—were similarly increased by both treatments (Figure 6A). Comparable effects were observed in sWAT and muscle (Supplementary Figure S6). Indeed, of the genes upregulated in the liver in HFD vs. CT (1166 genes, FC > 1.5, *p* < 0.05), 34% were downregulated by HIIT (395 genes, FC < −1.5, *p* < 0.05), with an additional 7% partially downregulated (81 genes, −1.5 < FC < −1.2, *p* > 0.05), whereas only two genes were further upregulated as compared with the HFD group.

The effects of EVs, although smaller (18% downregulated genes and 5% partially downregulated, with 23 upregulated), were comparable. The data for the sWAT and gastrocnemius were similar (Figure 6B). To analyze the extent to which the effects of HIIT and EV were overlapping, we created Venn diagrams comparing the genes upregulated by diet (HFD/CT) with those genes downregulated by the HIIT or EV treatments as compared with the untreated HFD mice. Similarly, Venn diagrams were created comparing genes that were downregulated in HFD mice and upregulated by the treatments. Hence, in the liver, of the total upregulated genes in HFD mice (100%), 11% were reverted by both treatments, whereas a further 23% were reverted only by HIIT and 7% only by EVs (Figure 6C). Similar percentages were observed in the case of downregulated genes in HFD livers. Overall, the liver was the tissue that responded best to the treatments. The effect of exercise was stronger than that of EV-miRNAs in all tissues (Figure 6C).

Searching for miRNAs regulating the expression of genes in the liver, sWAT, and muscle in obese mice through WebGestalt, we identified the GGGACCA_MIR133A_MIR133B matrix as a candidate in the liver (*p* = 0.14611), while ACACTCC_MIR122A (*p* = 0.16828) was the highest-ranking candidate in sWAT (Supplementary Table S4). Importantly, sWAT *miR-122* targets were downregulated in obese mice, and most of them were upregulated in EV mice that were treated with *miR-122* inhibitors (Figure 6D). Conversely, liver *miR-133* targets were upregulated in obese mice and decreased after treatment with EV-miRNAs including the *miR-133b* mimic (Figure 6E).

### 2.7. EV-miRNAs Regulate Hepatic Lipid Metabolism to Decrease Steatosis

**Figure 7:**
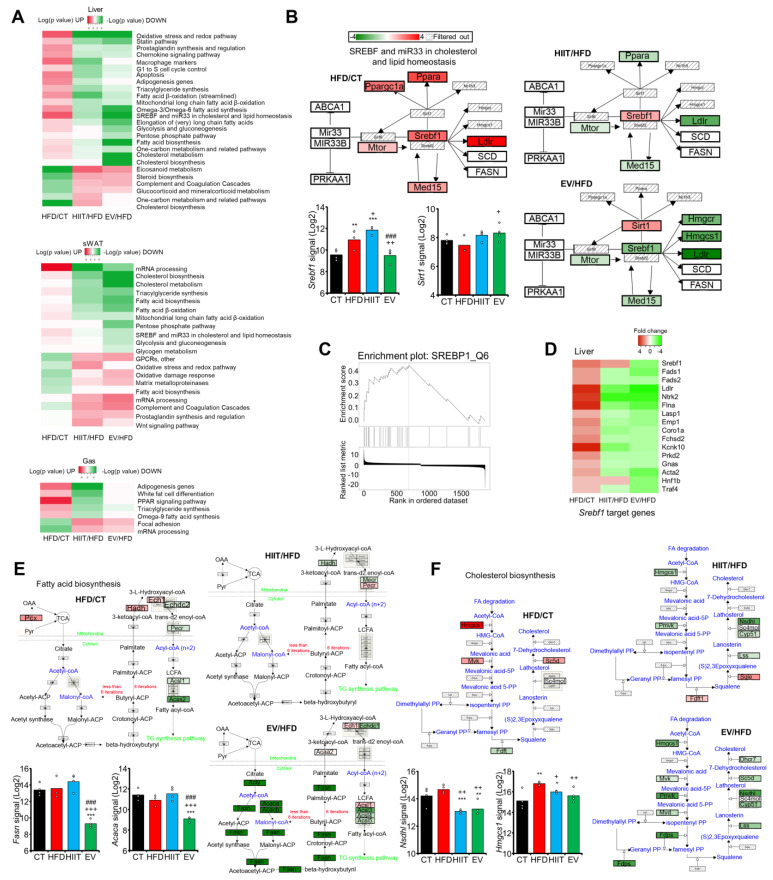
EV-miRNAs regulate hepatic lipid metabolism to decrease steatosis: (**A**) Heatmaps showing the log(*p*-value) of pathways enriched in upregulated (red, represented as −log(*p*-value)) or downregulated (green, represented as log(*p*-value)) genes in HFD/CT, HIIT/HFD, and EV/HFD. (**B**) The obesity-upregulated cholesterol and lipid homeostasis was normalized only by EV treatment in the liver. (**C**) Gene set enrichment analysis identified SREBP1 as a candidate transcription factor governing gene expression in EV livers. (**D**) SREBP1 target genes were decreased by EV treatment. (**E**) The fatty acid biosynthesis pathway was decreased specifically in EV livers. (**F**) Differentially enriched pathways with respect to the HFD group and sample signals of principal genes of interest in sWAT show a larger effect of EV-miRNAs on cholesterol biosynthesis. n = 4/group (**A**–**F**); * *p* < 0.05, ** *p* < 0.01, *** *p* < 0.005 with respect to the CT group; + *p* < 0.05, ++ *p* < 0.01, +++ *p* < 0.005 with respect to the HFD group; ### *p* < 0.005 between the HIIT and EV groups.

Finally, we compared pathway enrichment in the up- and downregulated DEGs in HFD mice vs. CT with the enrichment in the opposite DEGs in HIIT or EV vs. HFD (Figure 7A). We observed that the impact of diet on some downregulated hepatic pathways—such as the steroid biosynthesis pathway (e.g., *Hsd3b4* and *Hsd3b5*)—was similarly reverted by both treatments (Supplementary Figure S7A,B). The same was observed with upregulated pathways, such as oxidative stress response (e.g., *Sod3* and *Nfkb1*) (Supplementary Figure S7C,D), whereas HIIT was more effective in decreasing inflammation (e.g., macrophage markers) (Figure 7A) and normalized a range of other upregulated pathways, including the G1–S cell cycle control (e.g., *Ccnd1* and *Cdkn1a*) (Supplementary Figure S7E,F).

Remarkably, EV mice showed additional hepatic effects to those induced by HIIT. Only EVs—but not HIIT—significantly modulated the expression of genes related to cholesterol and lipid homeostasis (e.g., *Srebf1* and *Sirt1*) (Figure 7B). Accordingly, gene set enrichment analysis signaled SREBF1, which was significantly decreased, as a candidate transcription factor governing gene expression in the EV liver (Figure 7C,D). Moreover, EVs decreased fatty acid biosynthesis (e.g., *Fasn* and *Acaca*)—a pathway that was not significantly altered by diet—to levels even below those of the CT group (Figure 7E). All of these data may help explain how EV-miRNAs reduce hepatic steatosis (Figure 4G,H, Supplementary Figure S4D,E).

Likewise, enrichment analysis of sWAT revealed that both the HIIT and EV treatments had significant effects on upregulated pathways in HFD mice, including TG synthesis (e.g., *Mogat2* and *Dgat2*) (Supplementary Figure S7G,H). Moreover, as in the liver, EVs strongly decreased cholesterol biosynthesis (e.g., *Nsdhl* and *Hmgcs1*) (Figure 7F). Finally, the muscle showed the smallest number of transcriptomic changes, even in the HIIT group (Figure 7A). However, HIIT—but not EVs—reverted the increased expression of adipogenic genes (e.g., *Pparg* and *Foxo1*) detected in HFD mice (Supplementary Figure S7I,J).

Overall, these analyses indicate that both treatments exerted similar effects on several key pathways, with HIIT being more effective in the muscle, while EVs had important additional effects in the liver.

## 3. Discussion

The last several years have seen the arrival of promising weight-loss-inducing drugs, such as GIP and GLP-1 receptor agonists [41]. However, at present, lifestyle interventions such as diet and exercise remain the most cost-effective treatments available [42,43]. Hence, alternative therapies to facilitate weight loss or improve metabolic homeostasis in patients unable to fully exercise should be developed.

EVs have enormous potential as drug delivery vehicles due to their enhanced biocompatibility and reduced immunogenicity compared to alternative polymer-based carriers [44,45]. Several studies have previously explored the potential of native or modified EVs as therapeutic tools, most of them using EVs isolated from cell cultures—particularly MSCs or dendritic cells, which are themselves immunomodulatory and participate in tissue regeneration; hence, exosomes derived from them are expected to share some of these features [18,46,47]. Here, we chose to use EVs isolated from the plasma of control lean mice by ultracentrifugation—a technique that does not separate different types of vesicles (including microvesicles and exosomes) and may be contaminated with small amounts of lipoproteins that are also able to carry miRNAs [18,48]. However, we have previously shown the validity of this technique to transfer genetic material systemically and regulate target genes when the EVs thus isolated are transfected with specific siRNAs [20,21]. On the other hand, although native EVs from MSCs have been shown to exert beneficial effects, including for the treatment of complications of diabetes [18], a preparative study showed that, in our hands, native EVs from the plasma of control, sedentary mice did not affect the metabolic parameters in either lean or obese mice. Hence, we decided to leave the control obese group untreated to avoid having to also inject the lean CT and obese HIIT groups. Moreover, the administration of either native or transfected exosomes did not induce any detectable alterations in the animals’ behavior or wellbeing, suggesting that this is a safe treatment. In fact, similar treatments are currently being tested in humans in clinical practice. For instance, the clinical trial NCT03608631 uses mesenchymal-stromal-cell-derived exosomes transfected with KrasG12D siRNA to treat participants with pancreatic cancer with a KrasG12D mutation. Similarly, miravirsen is an *miR-122* antagomir used to treat hepatitis C [49].

Interestingly, as previously reported by others [13,50], our biodistribution study showed that exogenous EVs tended to accumulate in the liver and, indeed, it was there that we observed the strongest effects of the EV-miRNAs. EVs predominantly modified lipid and cholesterol metabolism, which was not unexpected, as the role of the selected miRNAs in cholesterol homeostasis has been previously demonstrated. Systemic inhibition of *miR-122* and *miR-192* using antagomirs or antisense oligonucleotides was described early on to reduce plasma cholesterol levels, increase hepatic FAO, and decrease hepatic fatty acid and cholesterol synthesis rates [51,52]. Our gene expression analysis indicated that we reproduced these effects with our administration mediated by EVs, using much lower doses of miRNA inhibitors. We injected 2.5 nmol/kg of each mimic or inhibitor, corresponding to about 15 µg/kg each, while other studies have used up to 1000-fold higher doses [51,52].

Remarkably, *miR-122* and *miR-192* are strongly associated with cardiometabolic diseases—particularly T2D and MAFLD—in humans [53,54,55]. Recent data show that *miR-122* promotes hepatic lipogenesis by targeting *Sirt1* [56], whereas its inhibition in vitro in 3T3-L1 cells decreased *Srebf1* [57]. We observed significantly increased *Sirt1* and decreased *Srebf1* in the liver and significantly increased *Sirt1* and a tendency to decrease *Srebf1* in the sWAT of EV mice—the two tissues that responded best to the EV-miRNAs treatment. *Sirt1* is an NAD+-dependent protein deacetylase that plays beneficial roles in hepatic lipid metabolism, oxidative stress, and inflammation by deacetylating transcription factors such as *Srebf1* [58]. These two factors may explain the improved metabolic profiles observed in EV mice—particularly the decreased hepatic steatosis and plasma TG levels, which may increase insulin sensitivity, thereby alleviating glucose intolerance. Accordingly, EV mice showed partially decreased basal hyperinsulinemia—a desirable feature for the treatment of obesity [59]. Additionally, we observed that *miR-122* inhibition had a stronger effect in the sWAT, resulting in upregulation of direct target genes with beneficial effects on obesity—including *Vav1*, whose knockout displayed increased fat content by decreasing *Sirt1* activity [60]; or *Ntrk3*, which promotes browning [61].

Finally, the *miR-133* family is consistently found to be increased in the blood after both acute and chronic exercise in humans [62], and it has also been involved in cardiac remodeling [63]. In particular, *miR-133a* was shown to be increased by exercise training, attenuating diabetes-induced cardiac injury in ovariectomized rats [64]. However, in our case, the obese mice failed to show patent cardiac alterations, so we were unable to observe any clear therapeutic effects of the treatments. Nevertheless, we observed an athlete’s heart phenotype in trained mice, pointing to the efficacy of the training. Importantly, we identified the *miR-133* family as regulators of gene expression in obese livers, and many of the upregulated target genes—some of them closely linked to the development of hepatic steatosis—were decreased by the EV-miRNA treatment, which may explain some of the beneficial effects observed. The protease *Senp2*, for instance, increases hepatic gluconeogenesis by ubiquitinating and decreasing 5’-AMP-activated protein kinase alpha (AMPKα); hence, downregulated *Senp2* may help explain the decrease in fasting glycemia in the EV mice [65]. On the other hand, *miR-133b* also directly targets enzymes involved in fatty acid biosynthesis, such as *Acaca* or *Acsl4*, whose inhibition may play a role in the decreased hepatic steatosis in EV mice [66,67].

With respect to the HIIT group, reduced body weight, fat mass, adipocyte size, and hepatic steatosis, along with increased insulin sensitivity in HFD mice, are all expected outcomes that have been described in some but not all reports, depending on the type of training or the duration of the high-fat feeding prior to starting training [68,69,70]. Interestingly, in contrast with the strong effects of the treatment with EV-miRNAs in decreasing the fatty acid and cholesterol biosynthesis pathways, HIIT did not affect the expression of key genes in these pathways—including *Acaca*, *Acacb*, and *Fasn*—and even increased hepatic *Srebf1* expression. We had previously observed that HIIT decreased *Foxo1* expression in both the liver and muscle of lean mice [20]. Here, in the context of obesity, we reproduced this effect in muscle, but not in the liver. Interestingly, HIIT reversed many of the gene expression alterations induced by diet in the muscle, whereas EV had a minimal effect in that tissue. These data may explain the improvements in metabolic flexibility and CRF that we observed in HIIT but not EV mice.

Hence, our data show that exercise enhanced metabolic flexibility, CRF, and insulin sensitivity, even under the continued administration of an obesogenic diet. Regarding the treatment with EV-miRNA, we reached the following conclusions: (i) administration of EV-miRNAs to obese mice being fed an HFD was unable to improve their metabolic flexibility or CRF; (ii) the treatment with EV-miRNAs increased insulin sensitivity and alleviated glucose tolerance to levels similar to those induced by exercise; (iii) both treatments exerted similar effects upon several key pathways, particularly in the liver and sWAT; and (iv) EVs had important additional effects in the liver by regulating fatty acid and cholesterol biosynthesis pathways. Overall, our results are consistent with the idea that a pharmacological treatment might be unable to fully mimic the beneficial effects of exercise [43,71]. However, manipulation of EV-miRNAs show promise for relieving obesity-associated metabolic deterioration in those individuals unable to adhere to a demanding exercise program.

## 4. Materials and Methods

### 4.1. Experimental Models

Fifteen-week-old mice were fed standard chow (n = 12) or an HFD (n = 36) for 10 weeks. Obese HFD mice were then distributed into three groups while being fed the same diet (n = 12/group): HFD mice remained untreated; HIIT mice were subjected to an HIIT protocol; EV mice were injected i.v. with plasma EVs from control mice transfected with *miR-122* and *miR-192* antimiRs and *miR-133b* mimics. The primary outcome to evaluate the success of the treatment was to obtain a reduction in the glucose AUC during a GTT. For biodistribution studies, control mice were injected i.v. with EVs labeled with ExoGlow-vivo EV Labeling Kits (System Biosciences) for in vivo analysis with an IVIS Imaging System and euthanatized at 6 h and 72 h after injection for microscopic analysis. Studies were performed at the School of Medicine Animal Facilities (University of Barcelona). The procedures were conducted in accordance with principles of laboratory animal care following EU Directive 2010/63/EU and approved by the Animal Research Committee of the University of Barcelona (register number: 46/18). C57BL/6J male mice were used throughout the study.

### 4.2. Indirect Calorimetry

An airtight one-lane treadmill with the CaloSys TSE Systems was used [20,21]. For the capacity test, mice were placed on the stationary treadmill for 5 min, followed by 2 min at 0.15 m/s, 2 min at 0.2 m/s, and increasing speed (0.0003 m/s^2^) until exhaustion. For the GTT, mice were placed on the stationary treadmill for 5 min to allow for gas equilibration. The chamber was then opened, and the mice were administered with the indicated glucose doses by oral gavage and replaced in the chamber.

### 4.3. Echocardiography and Electrocardiogram

Transthoracic echocardiographic studies were performed at least 12 h after the last training session in the HIIT group, and an ECG was obtained in vivo at the end of the experimental protocol by following previously described procedures [72].

### 4.4. EV Isolation, Characterization, Labeling, and Transfection

EVs were isolated from 500 μL of murine plasma, characterized, and transfected as described previously with 200 pmol each of the *miR-133b* mimic and *miR-122* and *miR-192* antimiRs (Exiqon-Qiagen, Hilden, Germany) Each transfection was enough for 2 injections, corresponding to 25 μg of EVs in 100 μL of PBS [20,21]. For biodistribution, aliquots of up to 250 μg of EVs were diluted with PBS and mixed with 2 μL of ExoGlow dye following the instructions of the ExoGlow-vivo EV Labeling Kit (System Biosciences, Palo Alto, CA, USA).

### 4.5. RNA Isolation and Gene Expression Analysis

Total RNA was extracted from frozen liver, sWAT, and gastrocnemius muscle tissues with the miRNeasy Mini Kit (Qiagen). Then, 150 ng of good-quality RNA samples (RIN > 9) was used for microarray hybridization. Fragmentation and biotin labelling of ss-cDNA was prepared according to the Affymetrix WT PLUS Reagent Kit user guide, using an automated system (Biomek FX System, Beckman Coulter, Brea, CA, USA). ss-cDNA was hybridized for 17 h at 45 °C on Clariom™ S HT murine array plates, using the automated GeneTitan System (Thermo Fisher Scientific, Waltham, MA, USA). We analyzed 4 biological replicates for each condition. The data were analyzed via Transcriptome Analysis Console 4.0 (Applied Biosystems, Waltham, MA, USA) using RMA analysis.

### 4.6. Statistical Analyses

Differences between groups were determined by one-way ANOVA with *t*-test analysis for the pairwise comparison of 3 or more groups with different numbers of values. Symbols indicate significance with respect to each control group.

## Data Availability

Microarray data have been deposited in the Gene Expression Omnibus (GEO) database (GSE199465).

## Supplementary Materials

The supporting information can be downloaded at: https://www.mdpi.com/article/10.3390/ijms232314920/s1.

## References

[1] González-Muniesa P., Mártinez-González M.A., Hu F.B., Després J.P., Matsuzawa Y., Loos R.J.F., Moreno L.A., Bray G.A., Martinez J.A. (2017). Obesity. Nat. Rev. Dis. Prim..

[2] Wang Y., Xu J., Wang Y., Hou H., Feng H., Yang H. (2021). An updated meta-analysis on the relationship between obesity and COVID-19 mortality. Metab. Clin. Exp..

[3] Deng T., Lyon C.J., Bergin S., Caligiuri M.A., Hsueh W.A. (2016). Obesity, Inflammation, and Cancer. Annu. Rev. Pathol..

[4] Saltiel A.R., Olefsky J.M. (2017). Inflammatory mechanisms linking obesity and metabolic disease. J. Clin. Investig..

[5] Goodpaster B.H., Sparks L.M. (2017). Metabolic Flexibility in Health and Disease. Cell Metab..

[6] Hivert M.-F., Christophi C.A., Franks P.W., Jablonski K.A., Ehrmann D.A., Kahn S.E., Horton E.S., Pollin T.I., Mather K.J., Perreault L. (2016). Lifestyle and Metformin Ameliorate Insulin Sensitivity Independently of the Genetic Burden of Established Insulin Resistance Variants in Diabetes Prevention Program Participants. Diabetes.

[7] Zaccardi F., O’Donovan G., Webb D.R., Yates T., Kurl S., Khunti K., Davies M.J., Laukkanen J.A. (2015). Cardiorespiratory fitness and risk of type 2 diabetes: A 23-year cohort study and a meta-analysis of prospective studies. Atherosclerosis.

[8] Brugnara L., Murillo S., Novials A., Rojo-Martínez G., Soriguer F., Goday A., Calle-Pascual A., Castaño L., Gaztambide S., Valdés S. (2016). Low physical activity and its association with diabetes and other cardiovascular risk factors. PLoS ONE.

[9] Joyner M.J., Green D.J. (2009). Exercise protects the cardiovascular system: Effects beyond traditional risk factors. J. Physiol..

[10] Astorino T.A., Schubert M.M. (2018). Changes in fat oxidation in response to various regimes of high intensity interval training (HIIT). Eur. J. Appl. Physiol..

[11] Karstoft K., Pedersen B.K. (2016). Skeletal muscle as a gene regulatory endocrine organ. Curr. Opin. Clin. Nutr. Metab. Care.

[12] Trovato E., Di Felice V., Barone R. (2019). Extracellular Vesicles: Delivery Vehicles of Myokines. Front. Physiol..

[13] Whitham M., Parker B.L., Friedrichsen M., Hingst J.R., Hjorth M., Hughes W.E., Egan C.L., Cron L., Watt K.I., Kuchel R.P. (2018). Extracellular Vesicles Provide a Means for Tissue Crosstalk during Exercise. Cell Metab..

[14] Garai K., Adam Z., Herczeg R., Banfai K., Gyebrovszki A., Gyenesei A., Pongracz J.E., Wilhelm M., Kvell K. (2021). Physical Activity as a Preventive Lifestyle Intervention Acts Through Specific Exosomal miRNA Species-Evidence From Human Short- and Long-Term Pilot Studies. Front. Physiol..

[15] Bartel D.P. (2018). Metazoan MicroRNAs. Cell.

[16] Ebert M.S., Sharp P.A. (2012). Roles for microRNAs in conferring robustness to biological processes. Cell.

[17] Chen X., Ba Y., Ma L., Cai X., Yin Y., Wang K., Guo J., Zhang Y., Chen J., Guo X. (2008). Characterization of microRNAs in serum: A novel class of biomarkers for diagnosis of cancer and other diseases. Cell Res..

[18] Castaño C., Novials A., Párrizas M. (2019). Exosomes and diabetes. Diabetes/Metab. Res. Rev..

[19] Thomou T., Mori M.A., Dreyfuss J.M., Konishi M., Sakaguchi M., Wolfrum C., Rao T.N., Winnay J.N., Garcia-Martin R., Grinspoon S.K. (2017). Adipose-derived circulating miRNAs regulate gene expression in other tissues. Nature.

[20] Castaño C., Kalko S., Novials A., Párrizas M. (2018). Obesity-associated exosomal miRNAs modulate glucose and lipid metabolism in mice. Proc. Natl. Acad. Sci. USA.

[21] Castaño C., Mirasierra M., Vallejo M., Novials A., Párrizas M. (2020). Delivery of muscle-derived exosomal miRNAs induced by HIIT improves insulin sensitivity through down-regulation of hepatic FoxO1 in mice. Proc. Natl. Acad. Sci. USA.

[22] Ying W., Gao H., Dos Reis F.C.G., Bandyopadhyay G., Ofrecio J.M., Luo Z., Ji Y., Jin Z., Ly C., Olefsky J.M. (2021). MiR-690, an exosomal-derived miRNA from M2-polarized macrophages, improves insulin sensitivity in obese mice. Cell Metab..

[23] Ying W., Riopel M., Bandyopadhyay G., Dong Y., Birmingham A., Seo J.B., Ofrecio J.M., Wollam J., Hernandez-Carretero A., Fu W. (2017). Adipose Tissue Macrophage-Derived Exosomal miRNAs Can Modulate In Vivo and In Vitro Insulin Sensitivity. Cell.

[24] Gallo-Villegas J., Aristizabal J.C., Estrada M., Valbuena L.H., Narvaez-Sanchez R., Osorio J., Aguirre-Acevedo D.C., Calderón J.C. (2018). Efficacy of high-intensity, low-volume interval training compared to continuous aerobic training on insulin resistance, skeletal muscle structure and function in adults with metabolic syndrome: Study protocol for a randomized controlled clinical trial. Trials.

[25] Wahl P., Bloch W., Proschinger S. (2021). The Molecular Signature of High-intensity Training in the Human Body. Int. J. Sport. Med..

[26] Gerosa-Neto J., Panissa V.L.G., Monteiro P.A., Inoue D.S., Ribeiro J.P.J., Figueiredo C., Zagatto A.M., Little J.P., Lira F.S. (2019). High- or moderate-intensity training promotes change in cardiorespiratory fitness, but not visceral fat, in obese men: A randomised trial of equal energy expenditure exercise. Respir. Physiol. Neurobiol..

[27] Metcalfe R.S., Fitzpatrick B., Fitzpatrick S., McDermott G., Brick N., McClean C., Davison G.W. (2018). Extremely short duration interval exercise improves 24-h glycaemia in men with type 2 diabetes. Eur. J. Appl. Physiol..

[28] Dela F., Ingersen A., Andersen N.B., Nielsen M.B., Petersen H.H.H., Hansen C.N., Larsen S., Wojtaszewski J., Helge J.W. (2019). Effects of one-legged high-intensity interval training on insulin-mediated skeletal muscle glucose homeostasis in patients with type 2 diabetes. Acta Physiol..

[29] Jelleyman C., Yates T., O’Donovan G., Gray L.J., King J.A., Khunti K., Davies M.J. (2015). The effects of high-intensity interval training on glucose regulation and insulin resistance: A meta-analysis. Obes. Rev. Off. J. Int. Assoc. Study Obes..

[30] Paramanantham A., Asfiya R., Das S., McCully G., Srivastava S. (2022). Extracellular Vesicle (EVs) Associated Non-Coding RNAs in Lung Cancer and Therapeutics. Int. J. Mol. Sci..

[31] Rajput A., Varshney A., Bajaj R., Pokharkar V. (2022). Exosomes as New Generation Vehicles for Drug Delivery: Biomedical Applications and Future Perspectives. Molecules.

[32] Cheng L., Yu P., Li F., Jiang X., Jiao X., Shen Y., Lai X. (2021). Human Umbilical Cord-Derived Mesenchymal Stem Cell-Exosomal MiR-627-5p Ameliorates Non-Alcoholic Fatty Liver Disease by Repressing FTO Expression. Hum. Cell.

[33] Niu Q., Wang T., Wang Z., Wang F., Huang D., Sun H., Liu H. (2022). Adipose-Derived Mesenchymal Stem Cell-Secreted Extracellular Vesicles Alleviate Non-Alcoholic Fatty Liver Disease via Delivering MiR-223-3p. Adipocyte.

[34] Duan Y., Luo Q., Wang Y., Ma Y., Chen F., Zhu X., Shi J. (2020). Adipose Mesenchymal Stem Cell-Derived Extracellular Vesicles Containing MicroRNA-26a-5p Target TLR4 and Protect against Diabetic Nephropathy. J. Biol. Chem..

[35] Wang J., Wu H., Peng Y., Zhao Y., Qin Y., Zhang Y., Xiao Z. (2021). Hypoxia Adipose Stem Cell-Derived Exosomes Promote High-Quality Healing of Diabetic Wound Involves Activation of PI3K/Akt Pathways. J. Nanobiotechnol..

[36] Liu Y., Li D., Liu Z., Zhou Y., Chu D., Li X., Jiang X., Hou D., Chen X., Chen Y. (2015). Targeted Exosome-Mediated Delivery of Opioid Receptor Mu SiRNA for the Treatment of Morphine Relapse. Sci. Rep..

[37] Sanz-de la Garza M., Rubies C., Batlle M., Bijnens B.H., Mont L., Sitges M., Guasch E. (2017). Severity of structural and functional right ventricular remodeling depends on training load in an experimental model of endurance exercise. Am. J. Physiol. Heart Circ. Physiol..

[38] Cavalcanti-de-Albuquerque J.P., Bober J., Zimmer M.R., Dietrich M.O. (2019). Regulation of substrate utilization and adiposity by Agrp neurons. Nat. Commun..

[39] Frayn K.N. (1983). Calculation of substrate oxidation rates in vivo from gaseous exchange. J. Appl. Physiol. Respir. Environ. Exerc. Physiol..

[40] Gonzalez-Franquesa A., Gama-Perez P., Kulis M., Szczepanowska K., Dahdah N., Moreno-Gomez S., Latorre-Pellicer A., Fernández-Ruiz R., Aguilar-Mogas A., Hoffman A. (2022). Remission of obesity and insulin resistance is not sufficient to restore mitochondrial homeostasis in visceral adipose tissue. Redox Biol..

[41] Samms R.J., Zhang G., He W., Ilkayeva O., Droz B.A., Bauer S.M., Stutsman C., Pirro V., Collins K.A., Furber E.C. (2022). Tirzepatide induces a thermogenic-like amino acid signature in brown adipose tissue. Mol. Metab..

[42] Mathus-Vliegen E.M.H. (2012). Obesity and the elderly. J. Clin. Gastroenterol..

[43] Wall C.E., Yu R.T., Atkins A.R., Downes M., Evans R.M. (2016). Nuclear receptors and AMPK: Can exercise mimetics cure diabetes?. J. Mol. Endocrinol..

[44] Prattichizzo F., Matacchione G., Giuliani A., Sabbatinelli J., Olivieri F., de Candia P., De Nigris V., Ceriello A. (2021). Extracellular vesicle-shuttled miRNAs: A critical appraisal of their potential as nano-diagnostics and nano-therapeutics in type 2 diabetes mellitus and its cardiovascular complications. Theranostics.

[45] de Abreu R.C., Fernandes H., da Costa Martins P.A., Sahoo S., Emanueli C., Ferreira L. (2020). Native and bioengineered extracellular vesicles for cardiovascular therapeutics. Nat. Rev. Cardiol..

[46] Milbank E., Dragano N.R.V., González-García I., Garcia M.R., Rivas-Limeres V., Perdomo L., Hilairet G., Ruiz-Pino F., Mallegol P., Morgan D.A. (2021). Small extracellular vesicle-mediated targeting of hypothalamic AMPKα1 corrects obesity through BAT activation. Nat. Metab..

[47] Kamerkar S., LeBleu V.S., Sugimoto H., Yang S., Ruivo C.F., Melo S.A., Lee J.J., Kalluri R. (2017). Exosomes facilitate therapeutic targeting of oncogenic KRAS in pancreatic cancer. Nature.

[48] Vickers K.C., Palmisano B.T., Shoucri B.M., Shamburek R.D., Remaley A.T. (2011). MicroRNAs are transported in plasma and delivered to recipient cells by high-density lipoproteins. Nat. Cell Biol..

[49] Bonneau E., Neveu B., Kostantin E., Tsongalis G.J., De Guire V. (2019). How close are miRNAs from clinical practice? A perspective on the diagnostic and therapeutic market. EJIFCC.

[50] Wiklander O.P.B., Nordin J.Z., O’Loughlin A., Gustafsson Y., Corso G., Mäger I., Vader P., Lee Y., Sork H., Seow Y. (2015). Extracellular vesicle in vivo biodistribution is determined by cell source, route of administration and targeting. J. Extracell. Vesicles.

[51] Esau C., Davis S., Murray S.F., Yu X.X., Pandey S.K., Pear M., Watts L., Booten S.L., Graham M., McKay R. (2006). miR-122 regulation of lipid metabolism revealed by in vivo antisense targeting. Cell Metab..

[52] Krutzfeldt J., Rajewsky N., Braich R., Rajeev K.G., Tuschl T., Manoharan M., Stoffel M. (2005). Silencing of microRNAs in vivo with “antagomirs”. Nature.

[53] Párrizas M., Brugnara L., Esteban Y., González-Franquesa A., Canivell S., Murillo S., Gordillo-Bastidas E., Cussó R., Cadefau J.A., García-Roves P.M. (2015). Circulating miR-192 and miR-193b are markers of prediabetes and are modulated by an exercise intervention. J. Clin. Endocrinol. Metab..

[54] Shah R., Murthy V., Pacold M., Danielson K., Tanriverdi K., Larson M.G., Hanspers K., Pico A., Mick E., Reis J. (2017). Extracellular RNAs are associated with insulin resistance and metabolic phenotypes. Diabetes Care.

[55] Pirola C.J., Gianotti T.F., Castaño G.O., Mallardi P., Martino J.S., Ledesma M.M.G.L., Flichman D., Mirshahi F., Sanyal A.J., Sookoian S. (2015). Circulating microRNA signature in non-alcoholic fatty liver disease: From serum non-coding RNAs to liver histology and disease pathogenesis. Gut.

[56] Long J.-K., Dai W., Zheng Y.-W., Zhao S.-P. (2019). miR-122 promotes hepatic lipogenesis via inhibiting the LKB1/AMPK pathway by targeting Sirt1 in non-alcoholic fatty liver disease. Mol. Med..

[57] Huang X.-Y., Chen J.-X., Ren Y., Fan L.-C., Xiang W., He X.-J. (2022). Exosomal miR-122 promotes adipogenesis and aggravates obesity through the VDR/SREBF1 axis. Obesity.

[58] Ding R.-B., Bao J., Deng C.-X. (2017). Emerging roles of SIRT1 in fatty liver diseases. Int. J. Biol. Sci..

[59] Kolb H., Stumvoll M., Kramer W., Kempf K., Martin S. (2018). Insulin translates unfavourable lifestyle into obesity. BMC Med..

[60] Qu P., Wang L., Min Y., McKennett L., Keller J.R., Lin P.C. (2016). Vav1 Regulates Mesenchymal Stem Cell Differentiation Decision Between Adipocyte and Chondrocyte via Sirt1. Stem Cells.

[61] Bové M., Monto F., Guillem-Llobat P., Ivorra M.D., Noguera M.A., Zambrano A., Sirerol-Piquer M.S., Requena A.C., García-Alonso M., Tejerina T. (2021). NT3/TrkC Pathway Modulates the Expression of UCP-1 and Adipocyte Size in Human and Rodent Adipose Tissue. Front. Endocrinol..

[62] Dufresne S., Rébillard A., Muti P., Friedenreich C.M., Brenner D.R. (2018). A review of physical activity and circulating miRNA expression: Implications in cancer risk and progression. Cancer Epidemiol. Biomark. Prev..

[63] Li N., Zhou H., Tang Q. (2018). miR-133: A Suppressor of Cardiac Remodeling?. Front. Pharmacol..

[64] Habibi P., Alihemmati A., Ahmadiasl N., Fateh A., Anvari E. (2020). Exercise training attenuates diabetes-induced cardiac injury through increasing miR-133a and improving pro-apoptosis/anti-apoptosis balance in ovariectomized rats. Iran. J. Basic Med. Sci..

[65] Dou X., Zhou W.-Y., Ding M., Ma Y.-J., Yang Q.-Q., Qian S.-W., Tang Y., Tang Q.Q., Liu Y. (2022). The protease SENP2 controls hepatic gluconeogenesis by regulating the SUMOylation of the fuel sensor AMPKα. J. Biol. Chem..

[66] Kim C.-W., Addy C., Kusunoki J., Anderson N., Deja S., Fu X., Burgess S.C., Li C., Ruddy M., Chakravarthy M. (2017). Acetyl CoA Carboxylase Inhibition Reduces Hepatic Steatosis but Elevates Plasma Triglycerides in Mice and Humans: A Bedside to Bench Investigation. Cell Metab..

[67] Duan J., Wang Z., Duan R., Yang C., Zhao R., Feng Q., Qin Y., Jiang J., Gu S., Lv K. (2022). Therapeutic targeting of hepatic ACSL4 ameliorates NASH in mice. Hepatology.

[68] Wang N., Liu Y., Ma Y., Wen D. (2017). High-intensity interval versus moderate-intensity continuous training: Superior metabolic benefits in diet-induced obesity mice. Life Sci..

[69] Marcinko K., Sikkema S.R., Samaan M.C., Kemp B.E., Fullerton M.D., Steinberg G.R. (2015). High intensity interval training improves liver and adipose tissue insulin sensitivity. Mol. Metab..

[70] Martinez-Huenchullan S.F., Ban L.A., Olaya-Agudo L.F., Maharjan B.R., Williams P.F., Tam C.S., Mclennan S.V., Twigg S.M. (2019). Constant-Moderate and High-Intensity Interval Training Have Differential Benefits on Insulin Sensitive Tissues in High-Fat Fed Mice. Front. Physiol..

[71] Booth F.W., Laye M.J. (2009). Lack of adequate appreciation of physical exercise’s complexities can pre-empt appropriate design and interpretation in scientific discovery. J. Physiol..

[72] Van de Water A., Verheyen J., Xhonneux R., Reneman R.S. (1989). An improved method to correct the QT interval of the electrocardiogram for changes in heart rate. J. Pharmacol. Methods.

