# Supplementary material for: Treatment with EV-miRNAs Alleviates Obesity-Associated Metabolic Dysfunction in Mice
Source: Int J Mol Sci. 2022 Nov 29;23(23):14920. doi: 10.3390/ijms232314920 (PMC9736074; doi:10.3390/ijms232314920)
Supplement: Supplementary file 1 [file ijms-23-14920-s001.zip › ijms-2019048-supplementary.pdf]

Supplementary data for:

Treatment with EV-miRNAs alleviates obesity-associated metabolic dysfunction in mice

Carlos Castaño[1,2], Aline Meza-Ramos[1], Montserrat Batlle[1,3], Eduard Guasch[1,3,4], Anna Novials[1,2,]* and Marcelina Párrizas[2,]*

[1]Instituto de Investigaciones Biomédicas August Pi i Sunyer (IDIBAPS), Barcelona-08036 (Spain); ccastano@clinic.recerca.cat
[2]Centro de Investigación Biomédica en Red de Diabetes y Enfermedades Metabólicas (CIBERDEM), Barcelona-08036 (Spain); anovials@recerca.clinic.cat
[3]Centro de Investigación Biomédica en Red de Enfermedades Cardiovasculares (CIBERCV), Barcelona-08036 (Spain); mbatlle@recerca.clinic.cat
[4]Cardiovascular Institute, Hospital Clinic, Barcelona-08036 (Spain); eguasch@clinic.cat

*Correspondence:
Anna Novials: anovials@recerca.clinic.cat
Marcelina Párrizas: badcell@gmail.com

Extended material and methods

Animal studies. All *in vivo* studies were performed at the School of Medicine Animal Facilities (University of Barcelona). Procedures were conducted in accordance with principles of laboratory animal care (EU Directive 2010/63/EU and local government guidelines) and approved by the Animal Research Committee of the University of Barcelona (register number: 46/18). C57BL/6J male mice were used throughout the study. Mice were fed standard chow or a high-fat diet (45% calories from fat, Open-Source Diets) for the periods indicated below.

Food intake and animal weight were controlled weekly. Glucose tolerance was determined by intraperitoneal (i.p.) GTT after 6 h fasting by injecting a dose of 2 g/Kg glucose. Pyruvate tolerance was determined by i.p. PTT in o/n fasted mice by injecting a dose of 1 g/Kg pyruvate. Insulin sensibility was determined by i.p. insulin tolerance test (ITT) after 6 h fasting by injecting a dose of 0.5 U/Kg insulin. In all cases, tail blood glucose was measured at 0, 15, 30, 60 and 120 min with reactive strips in a *NovaPro glucometer* (1, 2). Circulating TG levels were measured in tail blood with reactive strips in an *Accutrend GCT* glucometer after 6 h fasting. Insulin levels were determined from 5 µl plasma at the 0- and 15-min time points during the GTT with Mouse Insulin ELISA Kit (*Alpha Diagnostic International*). Plasma was obtained from 10 µl of tail blood centrifuged at 1,500 xg for 20 min (4ºC). The insulinogenic index was calculated as $\Delta Insulin_{(15\text{-}0)}/\Delta Glucose_{(15\text{-}0)}$, with the glucose (mg/dL) and insulin (ng/mL) values measured during the GTT.

At the sacrifice, liver, eWAT, sWAT, brown adipose tissue (BAT), gastrocnemius muscle, pancreas and heart were dissected, weighed and flash-frozen in liquid N_2 for posterior protein and RNA analyses. Tibia length was measured with a caliber. Hepatic NEFA and TG contents were measured after extraction with 3 M KOH and 65% ethanol with NEFA-HR(2) Kit and the GPO-POD Colorimetric Kit (*Spinreact*) respectively as described (1, 2).

Experimental models. 15-week-old mice were fed standard chow (n=12) or a high-fat diet (n=36) for 10 weeks. Obese HFD mice were then distributed into three groups while still being fed the same diet: HFD, HIIT and EV (n=12/group). HFD mice remained untreated for the next 4 weeks. HIIT mice were subjected to a HIIT protocol (1, 2). Training was performed in a six-lane treadmill and involved 2 min of running at 80% maximal running speed achieved in an initial exercise capacity test followed by 2 min of rest for a total 60 min with 25º inclination, 3 days per week. Running speed was increased 2 m/min every week for the next 4 weeks. This means that mice started running at 8 m/min on the first week and ended at 18 m/min on the fourth week. EV mice were injected i.v. through the tail vein with a 100 µl PBS suspension containing 25 µg EVs isolated from plasma of control mice and transfected with 100 pmol each of *miR-122* and *miR-192* antimiRs *and miR-133b* mimic. Injections took place biweekly during 4 weeks for a total of 8 injections. Mice were sacrificed 48 h after the last exercise session or EV injection. Two different cohorts of mice (n=6/group each time) were analyzed. For biodistribution studies, control mice were injected i.v. with a 100 µl PBS suspension containing 200 µg EVs isolated from mouse plasma and labeled with with ExoGlow for *in vivo* analysis with an IVIS Imaging System. Mice were sacrificed at 6 h and 72 h after the injection for microscopy analysis.

Indirect calorimetry. To perform a capacity test, mice were placed for 5 min in a stationary, airtight, one-lane treadmill with the CaloSys apparatus TSE Systems. This was followed by 2 min at 0.15 m/s, 2 min at 0.2 m/s, and increasing speed (0.0003 m/s^2 acceleration) until exhaustion, defined as the point at which mice remained for more than 10 s on the shockers delivering a 1.6 mA electric discharge that serves to encourage running. Final distance traversed and time spent running was recorded for each mouse at this point.

Gas exchange during a GTT was measured in a closed one-lane treadmill (1, 2). Each mouse was individually placed in the stationary treadmill chamber without inclination for 5 min to allow for gas equilibration. Chamber was then opened, and mice were orally administered with 1, 2, 4 or 6 g/kg glucose doses and placed again in the chamber. VO_2 and VCO_2 measurements were continued for the next 40 min. From these values, RER was determined as VCO_2/VO_2, dRER was determined as RER_{max}-RER_{min}, CHO was determined as $4.55xVCO_2$-$3.21VO_2$, and FAO was determined as $1.67xVO_2$-1.67CVO2 (3).

Echocardiography. Transthoracic echocardiographic studies were performed in all groups, at least 12 h after the last training session in the HIIT group. The procedure was performed under general anesthesia (inhaled isoflurane 1.5%) and a heating pad, and a phased-array probe 10S (4.5-11.5 Megahertz) attached to a commercially available system (Vivid Q, *GE Healthcare Ultrasound*). The M-mode spectrum was traced in a para-external long axis plane at the level of aortic valve and left atrial antero-posterior diameter was quantified. Left ventricular (LV) diameter (LVEDD), interventricular septum (IVS), and posterior wall (PW) thickness were measured at end-diastole at the level of the papillary muscles. All cardiac dimensions were tibia length-indexed to account for differences in body size. LV ejection fraction (LVEF) was estimated using previously validated formulas in rodents:

$$LVEF = \frac{LVEDD^2 - LVESD^2}{LVEDD^2}$$

LV diastolic function was estimated by the filling peak velocities E and A of the trans-mitral flow, and the E/A ratio was calculated (4). All measurements were obtained offline using a specific software package (EchoPac, *GE Healthcare*).

Electrocardiogram. An ECG was obtained *in vivo* at the end of the experimental protocol under inhaled anesthesia (isoflurane 1.5%) and a heating pad. A continuous lead II ECG was obtained and recorded for later offline analyses (PowerLab and LabChart v.8.1.2, *AD Instruments*). Analyses were performed blind to group assignment. After stabilization, the heart rate (beats/min), PR interval (ms), P-wave duration (ms), QRS duration (ms) and van der Water-corrected QT interval (QTc, ms) were automatically determined and averaged over a 2-minute period, manually reviewed for accuracy, and modified if needed (5).

EV isolation, characterization, labeling and transfection. EVs were isolated from 500 µl mouse plasma. Briefly, plasma was diluted with an equal volume of PBS and samples were sequentially centrifuged at 2,000 xg for 30 min (4ºC), 10,000 xg for 45 min (4ºC), filtered through a 0.22 µm syringe filter and ultracentrifuged o/n at 120,000 xg (4ºC) in a S110AT Rotor in a Sorvall MX 150 ultracentrifuge. Pellets were resuspended in PBS and ultracentrifuged again at 120,000 xg for 3 h (4ºC). The final pellets were resuspended in 100 µl PBS.

Total EV protein was quantified by Bradford Assay (*Sigma-Aldrich*) and complementary analyses by measuring the esterase activity known to be within exosomes were also performed in 5 µl of EVs in suspension by using Exocet Exosome Quantification Assay Kit (*System Biosciences*). Vesicle morphology was analyzed by negative staining in a TEM. Briefly, 30 µl EV samples diluted 1/10 to 1/20 with PBS were allowed to dry on top of formvar carbon-coated grids for 25 min and contrasted with 2% uranyl acetate for 2 min. Preparations were observed in a JEOL 1010 100 kV TEM. Particle number was determined by NTA with NanoSight LM10 equipment using different dilutions (1/10 to 1/50) and the following parameters: camera at 30 frames per second, camera level at 16, temperature between 21–25°C and video recording time 60 s. Nanosight Software analyzed raw data videos by triplicate. 10 µl EVs were resolved by 12% SDS-PAGE and transferred to a NTC membrane (Whatman). Anti-CD63 (H-193) (sc-15363, Santa Cruz Biotechnology) and Anti-CD9 (EPR2949) (ab92726, Abcam) were diluted

1/250 and 1/500 respectively in TBS (20mM Tris, 150mM NaCl, pH7.5) supplemented with 5% BSA and visualized by blotting with HRP-conjugated secondary anti-rabbit antibody (NA934V, GE Healthcare Bio-Sciences). Chemiluminiscence was detected by using the ECL Plus Reagents (GE Healthcare Bio-Sciences), in a LAS4000 Lumi-Imager (Fuji Photo Film Inc.). EVs were fluorescently labeled with ExoGlow-vivo EV Labeling Kit (*System Biosciences*). Aliquots up to 250 µg EVs were diluted with PBS and mixed with 2 µl ExoGlow-vivo dye. Incubation was stopped by adding ExoQuick-TC reagent by incubating o/n (4ºC) and ultracentrifuging at 13,000 xg for 10 min. The final pellet was resuspended in 100 µl PBS.

EVs (50 µg) were transfected with 200 pmol/each *miR-133b* mimic and *miR-122* and *miR-192* antimiRs (*Exiqon-Qiagen*) by using Exo-Fect Exosome Transfection Reagent (*System Biosciences*). Each transfection was enough for 2 injections, corresponding to 25 µg EVs in 100 ul PBS (1, 2).

Histochemistry and immunofluorescence. Tissues were fixed with 4% PFA o/n. Liver and pancreas were equilibrated in 30% sucrose and embedded in OCT, whereas adipose tissues and heart were embedded in paraffin. 10 µm sections were obtained with a CM 1950 cryostat or a RM2135 microtome respectively and applied to poly-lysine coated slides. Oil Red O and Hematoxylin & Eosin stainings were performed by following the protocols at IHCWorld (http://www.ihcworld.com). Liver sections were mounted in Aquatex mounting media (*Merck*) and adipose sections were mounted in Pertex mounting medium (*HistoLab)*. Images were obtained with a BX41TF trinocular microscope.

Insulin area was determined in islets of pancreatic sections from 2 different levels (>150 mm apart) for each pancreas. Briefly, dewaxed sections were rehydrated, permeabilized with 1% Triton X-100 (*Sigma-Aldrich*) and blocked with 3% BSA (*Sigma-Aldrich*). Sections were then incubated o/n at 4ºC in a wet chamber with 1:1000 guinea pig anti-insulin (*Dako*) and 1:1000 mouse anti-glucagon (*Sigma-Aldrich*) in antibody diluent (*Dako*) followed by 1:250 donkey anti-guinea pig-Cy5 (*Jackson ImmunoResearch*) and 1:250 goat Alexa Fluor 488 anti-mouse (*Jackson ImmunoResearch*) secondary antibodies respectively for 2 h at RT in the dark. Macrophage infiltration was determined in sWAT sections incubated with 20 µg/ml Proteinase K solution in TE buffer for 3 min at RT and rinsed with PBS before permeabilization. Sections were then incubated in same conditions with 1:500 rabbit anti-F4/80 (*Abcam*) followed by 1:250 donkey anti-rat-Cy3 (*Jackson ImmunoResearch*). All slides were then washed with PBS and nuclei were stained with 1:500 Hoestch (*Sigma-Aldrich*) in PBS for 3 min, and the preparations were mounted with Dako fluorescence mounting medium (*Dako*).

LV and right ventricular (RV) fibrosis was assessed in histological preparations as previously described (4). Briefly, after euthanasia, hearts were embedded into paraffin. A mid-papillary ventricular section was subsequently obtained and stained with Sirius red staining to identify collagen deposition. A single microphotograph, including the whole section, was obtained (Panoramic Viewer, *3DHISTECH*). Myocardial fibrosis was semiautomatically quantified with ImageJ (NIH), and results given as percentages. Perivascular and epicardial fibrosis were excluded from the analysis.

To study biodistribution of ExoGlow-labeled EVs, the same tissues plus the gastrocnemius muscle were embedded in OCT to obtain 10 µm sections. Nuclei were stained with 1:500 Hoestch in PBS for 3 min and were mounted with Dako fluorescence mounting medium. All images were collected using TCS SPE confocal microscope and processed with Adobe Photoshop 7.0 and ImageJ FIJI (1, 2).

Western blotting. Total protein was extracted using RIPA lysis buffer from frozen tissue samples after powdering them with a ceramic mortar and pestle in liquid N_2. Appropriate protease and phosphatase inhibitors were added fresh to the lysis buffer.

50 µg quantified by Bradford Assay (*Sigma-Aldich*) were resolved by 7.5% Mini-PROTEAN TGX Precast Protein Gels (*Bio-Rad*) and transferred to a NTC membrane (*Whatman*). After 5% BSA blocking, rabbit anti-FAS (*Abcam*) antibody was diluted 1:1000 in 1% TBS-BSA and visualized by blotting with 1:5000 HRP-conjugated secondary anti-rabbit antibody (*GE Healthcare*). Chemiluminiscence was detected with Pierce ECL Western Blotting Substrate (*Thermo Fisher*) in a LAS4000 Lumi-Imager.

RNA isolation and gene expression analysis. Total RNA was extracted from frozen liver, sWAT and gastrocnemius muscle with miRNeasy Mini Kit (*Qiagen*), treated with DNAse, and quantified by absorbance with Nanodrop. 250 ng RNA was used to analyze size and integrity with TS4200. 150 ng of good quality RNA samples (RIN >9) with a Ratio 260/280 = 1.8-2 were used for microarray hybridization. Fragmentation and Biotin labelling of ss-cDNA was prepared according to Affymetrix WT PLUS Reagent Kit user guide, using an automated system (Biomek FX System, Beckman Coulter). Following fragmentation and terminal labeling, ss-cDNA was hybridized for 17hr at 45ºC on Clariom™ S HT, mouse array plate, using the automated GeneTitan System (*Thermo Fisher Scientific*), which includes the hybridization oven, Fluidic Station and Scanner. We analyzed 3 tissues in 4 groups of mice (CT, HFD, HIIT, EV) for a total of n=12 samples, performing quadruplicates for a total of n=48 microarray hybridizations.

The data was analyzed with Transcriptome Analysis Console 4.0 (*Applied Biosystems*) using RMA analysis. PCAs, scatter plots, hierarchical clusterings and pathway enrichment analysis were performed with the same software. Gene set enrichment analysis was performed with WebGestalt (http://www.webgestalt.org). Heat maps of pathways identified by enrichment analysis of genes were created with heatmapper.ca. Venn's diagrams for comparing up lists of genes were created with Venny 2.1 (https://bioinfogp.cnb.csic.es).

Statistical analyses. Differences between groups were determined by either t-test analysis when only two groups were compared or by One-way ANOVA with t-test analysis for the pair wise comparison of 3 or more groups with different number of values. Symbols indicate significance with respect to each control group.

References

1. Castaño C, Kalko S, Novials A, Párrizas M (2018) Obesity-associated exosomal miRNAs modulate glucose and lipid metabolism in mice. *Proc Natl Acad Sci USA*. **115**:12158-12163.
2. Castaño C, Mirasierra M, Vallejo M, Novials A, Párrizas M (2020) Delivery of muscle-derived exosomal miRNAs induced by HIIT improves insulin sensitivity through down-regulation of hepatic FoxO1 in mice. *Proc Natl Acad Sci USA* **117**:30335–30343.
3. Frayn KN (1983) Calculation of substrate oxidation rates in vivo from gaseous exchange. *J Appl Physiol* **55**:628–634.
4. Sanz-de la Garza M, et al. (2017) Severity of structural and functional right ventricular remodeling depends on training load in an experimental model of endurance exercise. *Am J Physiol Heart Circ Physiol* **313**:H459–H468.
5. Van de Water A, Verheyen J, Xhonneux R, Reneman RS (1989) An improved method to correct the QT interval of the electrocardiogram for changes in heart rate. *J Pharmacol Methods* **22**:207–17.

Supplementary Table S1. Modified pathways identified by enrichment analysis of differentially expressed genes in liver of HFD vs. CT mice.

Pathway	Up	Representative Up genes	Down	Representative Down genes	p-value
Ribosomal proteins	1	Rps6ka1	37	Rps18, Rpl3	0.0000
Eicosanoid metabolism	2	Cyp2c38	7	Cyp2c40,Cyp4f14	0.0000
PPAR signaling pathway	16	Cd36, Fabp4	5	Plin1, Fabp5	0.0000
Cholesterol biosynthesis	0	-	8	Sqle, Idi1	0.0000
Steroid biosynthesis	2	Hsd17b4	5	Hsd3b4, Hsd3b3	0.0000
GPCRs, other	3	Adora1, Ednra	0	-	0.0000
Macrophage markers	5	Cd14, Cd68	1	F3	0.0000
IL-5 signaling pathway	16	Nfkb1, Itgam	0	-	0.0001
SREBF and miR33	6	Srebf1, Ldlr	0	-	0.0003
Oxidative stress response	9	Sod3, Gpx3	0	-	0.0004
IL-3 signaling pathway	17	Foxo1, Hspb1	2	Tec, Gnb2l1	0.0006
Complement activation	1	C1qb	5	C8a, C2	0.0018
IL-7 signaling pathway	9	Stat1, Bax	1	Irs2	0.0027
TCA cycle	7	Ogdh, Mdh2	1	Pdk3	0.0031
One-carbon metabolism	6	Chpt1, Cept1	5	Csad, Bhmt2	0.0031
Adipogenesis genes	17	Id3, Cebpb	4	Bmp1, Lifr	0.0040
T cell receptor signaling	20	Dock2, Rasgrp2	1	Cd4	0.0040
IL-6 signaling pathway	15	Foxo1, Eif4ebp1	2	Tec, Eif4e	0.0049
Insulin signaling	21	Pik3r1, Gsk3b	3	Irs2, Xbp1	0.0055
Focal adhesion	25	Actg1, Rac2,	2	Egfr, Ppp1r12a	0.0064
Apoptosis	13	Bak1, Jun	1	Irf6	0.0068
B cell receptor signaling	21	Pdk2, Pip4k2a	2	Rasgrp3, Tec	0.0078
Triacylglyceride synthesis	5	Mogat1, Pnpla2	1	Lipc	0.0096
ω-3/ω-6 fatty acid synthesis	5	Fads2, Pla2g6	0	-	0.0104
Prostaglandin synthesis	7	Anxa1, Anxa2	0	-	0.0121
Fatty acid beta-oxidation	7	Cpt1a, Fabp4	0	-	0.0144
Oxidative damage	6	C1qb, Cdkn1a	2	Hc, C2	0.0181
Statin pathway	4	Apoc2, Apoa4	1	Lipc	0.0213
Toll-like receptor signaling	14	Tlr2, Cxcl10	1	Tbk1	0.0250
TNF-alpha NF-kB signaling	16	Fkbp5, Usp2	8	Ktn1, Traf5	0.0304
IL-2 signaling pathway	9	Il2rg, Pik3r1	3	Irs2, Eif4e	0.0331
Glycolysis	5	Pdha1, Slc2a1	2	Aldoc, Pdk3	0.0350

Supplementary Table S2. Modified pathways identified by enrichment analysis of differentially expressed genes in sWAT of HFD vs. CT mice.

Pathway	Up	Representative Up genes	Down	Representative Down genes	p-value
Ribosomal proteins	2	Rps6ka2	57	Rps18, Rpl3	0.0000
GPCRs, other	3	Adora1, Fzd5	8	Xcr1, Ccr7	0.0000
Focal adhesion	39	Col4a1, Gsk3b	22	Rac2, Txk,	0.0000
Chemokine signaling	19	Foxo3, Pik3cb	40	Arrb2, Cxcl16	0.0000
B cell receptor signaling	18	Ccnd2, Gab2	33	Sla2, Bcl10	0.0000
T cell receptor signaling	12	Gab2, Rasgrp2	31	Lime1, Map4k1	0.0003
Matrix metalloproteinases	6	Timp3, Timp4	8	Mmp2 Timp2	0.0004
IL-3 signaling pathway	12	Ptpn11,R xra	20	Pik3cd, Tec,	0.0032
Mapk signaling pathway	26	Dusp1, Tmem37	20	Tgfbr2, Arrb2	0.0033
Adipogenesis genes	26	Pparg, Lep	14	Creb1, Ppara,	0.0039
IL-2 signaling pathway	6	Pik3cb, Foxo3	19	Il2rb, Lck	0.0053
Macrophage markers	0	-	6	Cd83, Cd86	0.0055
IL-5 signaling pathway	5	Ptpn11, Unc119	18	Csf2rb, Prkcb	0.0058
Complement activation	1	C6	7	C1qb, C1qa	0.0092
Omega-9 fatty acid synthesis	1	Acsl1	6	Fads1, Fasn	0.0100
G protein signaling pathways	17	Prkar2b, Pde7b	11	Prkacb, Gna13	0.0109
TCA cycle	1	Idh2	11	Pdha1, Pdk1	0.0114
Na/K-ATPase/Src signaling	14	Mapk6, Map2k3	13	Rac2, Txk	0.0142
Insulin signaling	28	Trib3, Foxo1	16	Irs1, Pik3cg	0.0156
SREBF and miR33	3	Nr1h3, Ldlr	3	Sirt1, Ppara	0.0265
Oxidative damage response	3	Cdkn1a, Tnk2	11	Pcna, C1qb,	0.0275
ω-3/ω-6 fatty acid synthesis	3	Acsl1, Pla2g5	4	Acot1, Acot2	0.0329
Prostaglandin synthesis	7	Anxa5, Anxa1	4	Anxa3 ,Hsd11b1	0.0375
Apoptosis	5	Ikbkg, Jun	18	Myc, Bcl2	0.0461

Supplementary Table S3. Modified pathways identified by enrichment analysis of differentially expressed genes in gastrocnemius muscle of HFD vs. CT mice.

**Pathway**	**Up**	**Representative Up genes**	**Down**	**Representative Down genes**	**p-value**
Fatty acid β-oxidation	11	Acadm, Cpt2	1	Acsl3	0.0000
Fatty acid biosynthesis	4	Hadh, Acaa2	5	Acaca, Acly	0.0000
LCFA β-oxidation	7	Acadl, Hadh	2	Acsl3, Pecr	0.0000
ω-9 fatty acid synthesis	2	Acot1, Acot2	4	Elovl6, Scd2	0.0000
Inflammatory response	0	-	7	Cd40, Cd86	0.0004
PPAR signaling pathway	10	Fabp3, Cd36	2	Scd2, Acsl3	0.0005
Adipogenesis genes	10	Lipe, Pparg	5	Igf1, Srebf1	0.0019
Cholesterol metabolism	3	Acat1, Acot1	6	Fasn, Sqle	0.0020
Hypertrophy model	2	Ankrd1, Hbegf	3	Il1r1, Nr4a3	0.0022
ID signaling pathway	0	-	7	Id4, Myf6	0.0121
GPCRs, other	3	Adora1, Olfr23	0	-	0.0144
SREBF and miR33	0	-	3	Hmgcr, Ldlr	0.0224
Mapk signaling pathway	6	Dusp1, Acvr1c	8	Il1r1, Egfr	0.0249
Toll-like receptor signaling	4	Nfkbia, Lbp	6	Pik3cg, Pik3r3	0.0278
Complement cascade	2	Cd59a,Serpine1	5	Plau, F12	0.0296
G1 to S cell cycle control	3	Ccnd2, E2f4	4	Mcm2, Mcm4	0.0296
Focal adhesion	6	Ddit4, Angpt1	18	Col5a3, Itga11	0.0374
ω-3/ω-6 fatty acid synthesis	2	Acot1, Acot2	1	Acsl3	0.0462
Matrix metalloproteinases	2	Mmp12, Timp4	2	Mmp14, Mmp27	0.0495

Supplementary Table S4. Candidate miRNA regulating liver, sWAT and gastrocnemius muscle expression in obese HFD mice, identified by WebGestalt.org.

HFD/CT Liver candidate miRNAs

Gene Set	Size	Leading Edge Nº	P Value	FDR
CAGGTCC_MIR492	132	3	0.000	0.011
TATCTGG_MIR488	104	29	0.163	0.295
ATACCTC_MIR202	302	99	0.166	0.297
TGCAAAC_MIR452	191	56	0.142	0.297
GGGACCA_MIR133A_MIR133B	283	107	0.146	0.299
GTGTCAA_MIR514	121	25	0.175	0.299
CATTTCA_MIR203	456	168	0.128	0.299
TCCGTCC_MIR184	21	18	0.206	0.300
TTGGAGA_MIR5155P_MIR519E	235	86	0.163	0.300
CAGTGTT_MIR141_MIR200A	516	197	0.109	0.301

HFD/CT sWAT candidate miRNAs

Gene Set	Size	Leading Edge Nº	P Value	FDR
ACACTCC_MIR122A	128	19	0.168	0.306
GTGCAAT_MIR25_MIR32_MIR92_MIR363_MIR367	506	119	0.138	0.308
CAGGTCC_MIR492	132	45	0.123	0.309
ACATTCC_MIR1_MIR206	474	118	0.126	0.310
GTAAACC_MIR2995P	83	29	0.108	0.310
TGCACTG_MIR148A_MIR152_MIR148B	476	127	0.136	0.310
TCTCTCC_MIR185	230	42	0.130	0.310
CTCTATG_MIR368	53	19	0.132	0.310
ACTTTAT_MIR1425P	485	106	0.121	0.310

HFD/CT Gas candidate miRNAs

Gene Set	Size	Leading Edge Nº	P Value	FDR
CCATCCA_MIR432	120	3	0.002	0.196
CAGGTCC_MIR492	132	37	0.007	0.216
ATGCACG_MIR517B	42	16	0.009	0.324
GCAAGAC_MIR431	68	14	0.022	0.389
GTGCCAA_MIR96	469	109	0.078	0.691
GTACTGT_MIR101	453	191	0.081	0.706
ATACTGT_MIR144	310	148	0.073	0.729
GGGATGC_MIR3245P	87	24	0.124	0.730
ACCAATC_MIR509	64	25	0.100	0.737
AACTGGA_MIR145	396	133	0.058	0.757

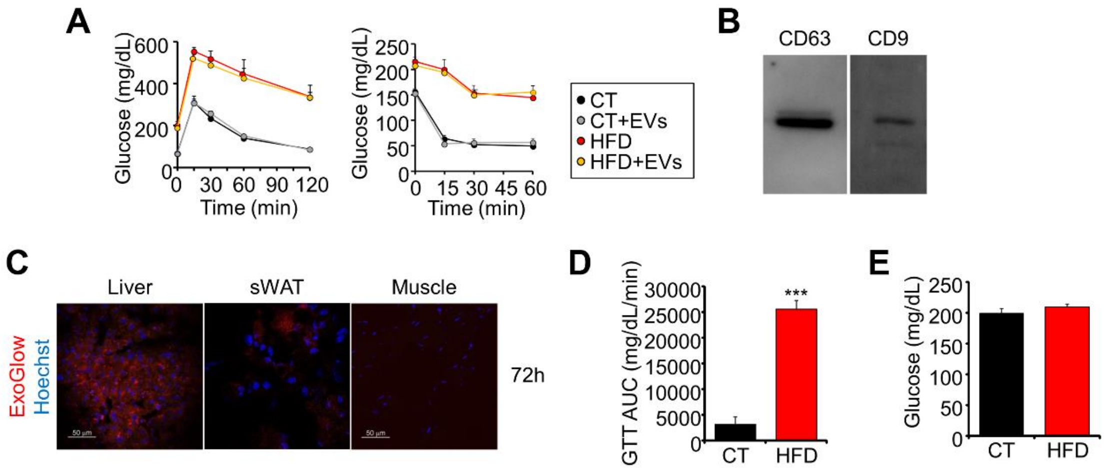

Supplementary Figure S1. Experimental design, exogenous EVs biodistribution and characterization of obese mice. (A) Four weeks treatment with native EVs isolated from the plasma of control mice do not induce metabolic changes in lean or obese HFD mice as determined by GTT (left panel) or ITT (right panel). **(B)** Plasma EVs used for treatment were characterized by western blot of membrane markers CD63 and CD9. **(C)** 72h after injection of ExoGlow-labelled EVs, fluorescence is detected in the liver, sWAT and gastrocnemius muscle by confocal microscopy. **(D, E)** After 10 weeks high-fat feeding, mice are glucose intolerant **(D)**, although they have not yet developed fasting hyperglycemia **(E)**. n=3 (CT), n=4 (HFD) (A); n=3 (B, C); n=6 (CT), n=18 (HFD) (D, E). ***$p<0.005$ with respect to CT group.

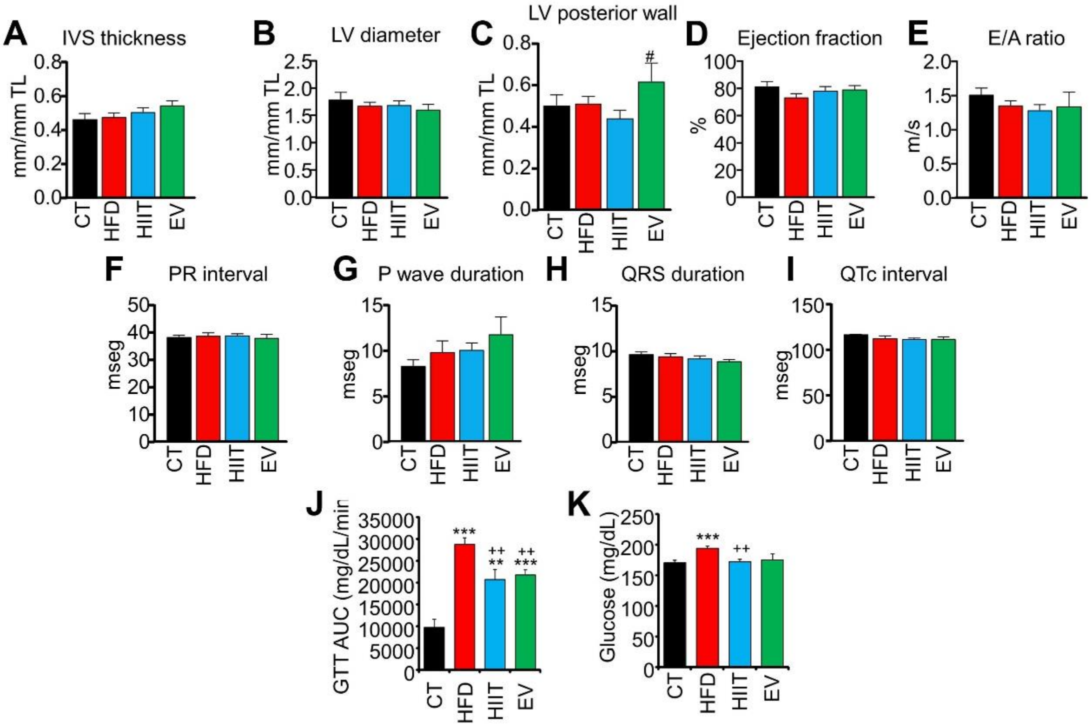

Supplementary Figure S2. HIIT and EV-miRNAs improve the metabolic profile of HFD mice but only HIIT enhances CRF and promotes cardiac remodeling. (A-E) Echocardiographic and ECG characterization of HFD, HIIT and EV mice. Echocardiographic data show no differences between the treatment groups and the CT of HFD groups in interventricular septum thickness **(A)**, left ventricular diameter **(B)**, LV posterior wall, **(C)**, ejection fraction **(D)** and E/A ratio **(E)**. **(F-I)** No significant differences were found between groups for PR **(F)**, P-wave **(G)**, QRS duration **(H)** and QTc interval **(I)**. **(J, K)** Both HIIT and EV improve glucose tolerance **(J)** and HIIT also significantly decrease fasting plasma glucose levels **(K)**. n=6/group (A-J). **p<0.01, ***p<0.005 with respect to CT group. $^{++}$p<0.01 with respect to HFD group. $^{\#}$p<0.05 between HIIT and EV groups.

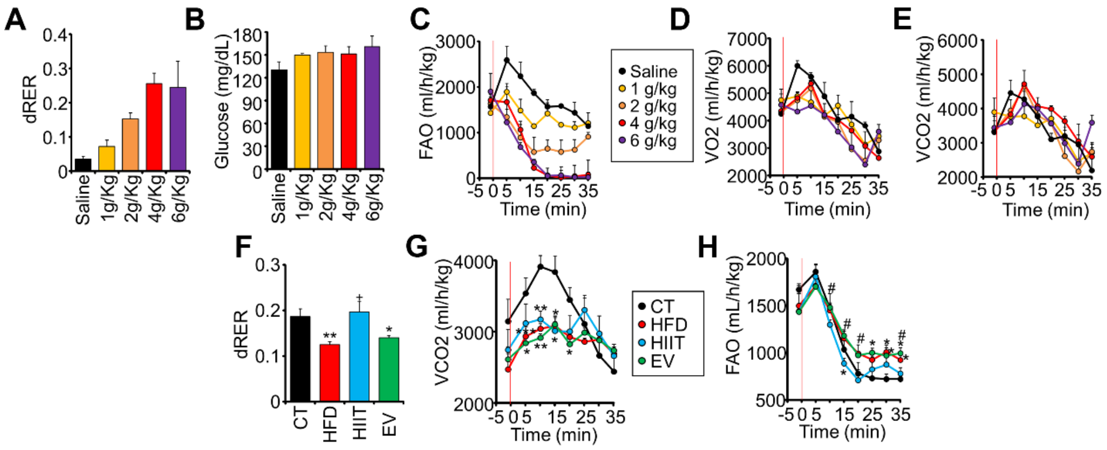

Supplementary Figure S3. HIIT and EV-miRNAs improve glucose tolerance through different mechanisms. (A, B) Oral administration of glucose to CT lean mice immediately and dose-dependently increases respiratory exchange ratio as measured by calorimetry **(A)**, while maintaining glycemia within normal levels at the end of the test **(B)**. **(C-E)** Glucose administration dose-dependently decreases fat oxidation **(C)**, as calculated from the values of O_2 consumption **(D)** and CO_2 production **(E)** during the test. **(F)** Untreated HFD mice show lower respiratory exchange ratio values than CT mice in response to glucose administration, and this is normalized by HIIT but not EV group. **(G, H)** Obese mice shown lower CO_2 production **(G)** and increased fat oxidation **(H)** than lean CT mice during the test, and this was not reverted by neither HIIT nor EV groups. Red line in graph indicates the time of glucose administration. n=2/group (A-E), n=5/group (F-M). *$p<0.05$, **$p<0.01$, ***$p<0.005$ with respect to CT group. $^{+}p<0.05$ with respect to HFD group. $^{\#}p<0.05$ between HIIT and EV groups.

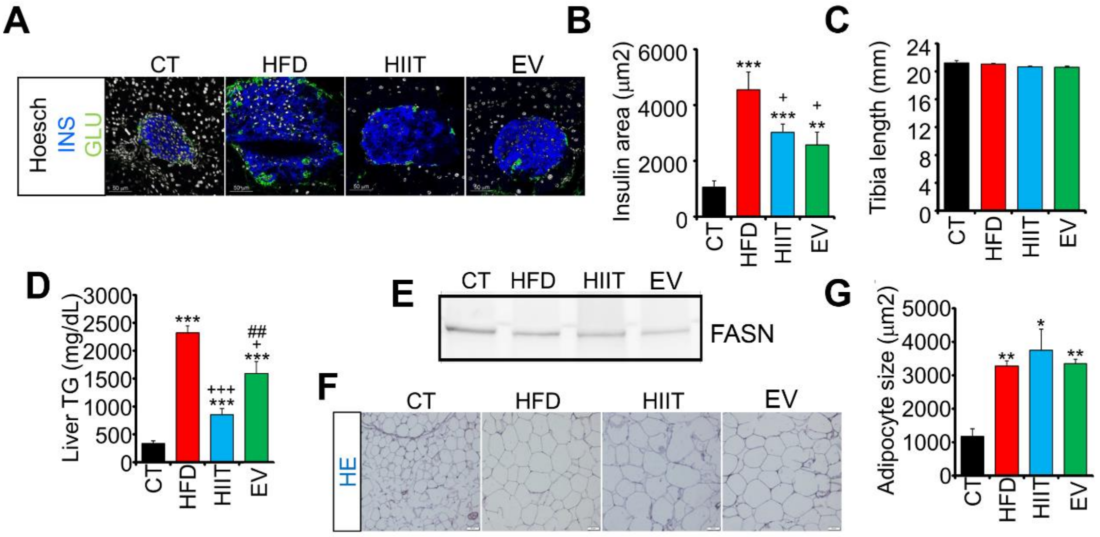

Supplementary Figure S4. Treatment with EV-miRNAs improves hepatic insulin sensitivity and steatosis. (A, B) Both HIIT and EV treatments show decreased pancreatic islet size **(A)** and insulin area **(B)**. **(C)** Tibia length shows comparable animal size between groups. **(D)** Untreated HFD mice show high levels of hepatic steatosis as determined by the quantification of liver triglycerides. **(E)** Western blot shows deceased FASN abundance after EV treatment. **(F, G)** Neither HIIT nor EV treatments affect adipocyte size as determined by Hematoxylin & Eosin staining in the eWAT. n=2/group (A, B, E, F, G), n=6/group (C, D). *p<0.05, **p<0.01, ***p<0.005 with respect to CT group. $^{+}$p<0.05, $^{+++}$p<0.005 with respect to HFD group. $^{\#\#}$p<0.01 between HIIT and EV groups.

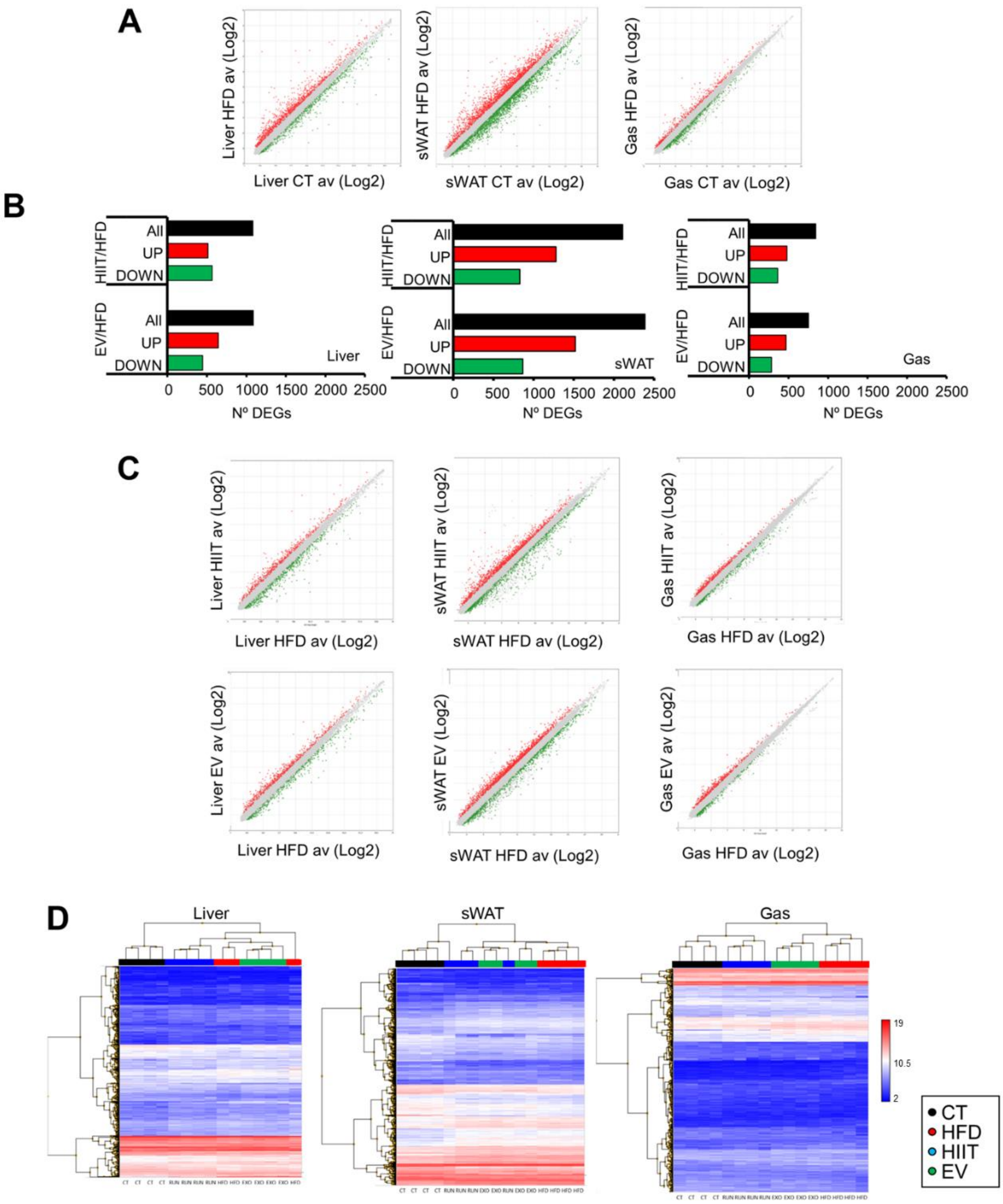

Supplementary Figure S5. HIIT and EV-miRNAs affect the hepatic, sWAT and gastrocnemius muscle expression profile of obese mice. (A) Scatter plots of genes in liver, sWAT and gastrocnemius muscle significantly altered in HFD mice at least 1.5-fold in each direction, $p<0.05$. **(B, C)** Number of significantly upregulated or downregulated genes **(B)** and scatter plots of genes modulated in the same tissues by HIIT and EV treatments with respect to HFD mice **(C)**. **(D)** Heatmaps showing hierarchical clustering of HFD/CT differentially expressed genes comparing CT, HFD, HIIT, and EV groups. n=4/group (A-D).

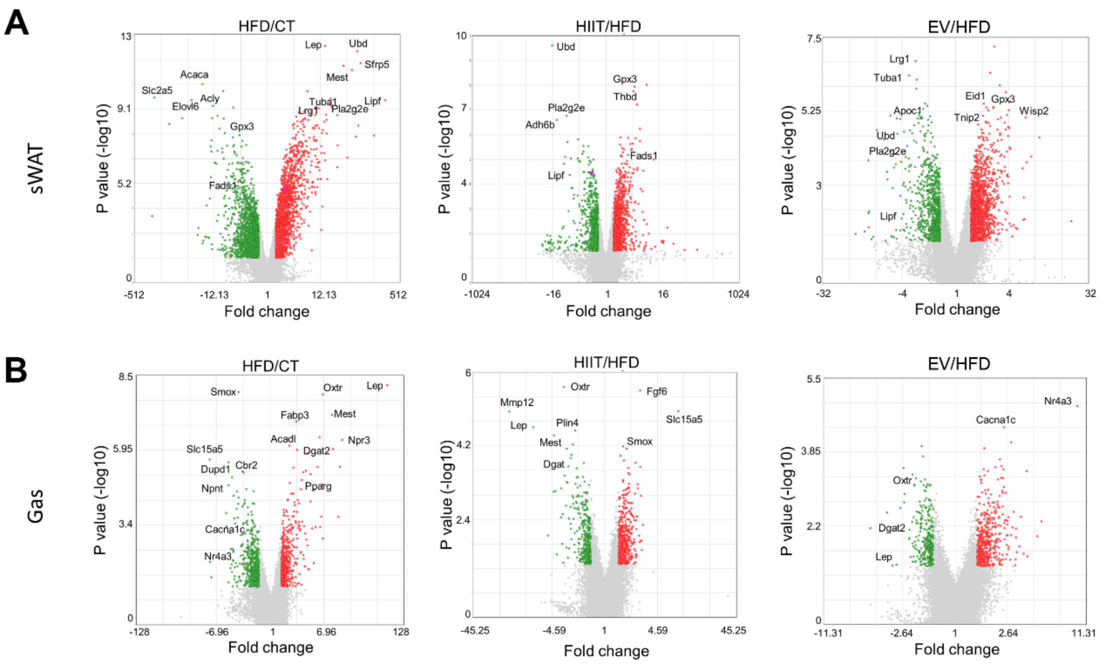

Supplementary Figure S6. HIIT and EV-miRNAs partially reverse the alterations induced by diet. (A and B) Volcano plots depicting expression changes in sWAT **(A)** and gastrocnemius muscle **(B)**. Obese HFD mice were compared with CT mice to determine the effects of the diet upon gene expression **(left panels)**. The effects of HIIT or EV treatments upon obese mice were determined by comparing treated mice with obese untreated mice **(middle and right panels)**. n=4/ group (A-B).

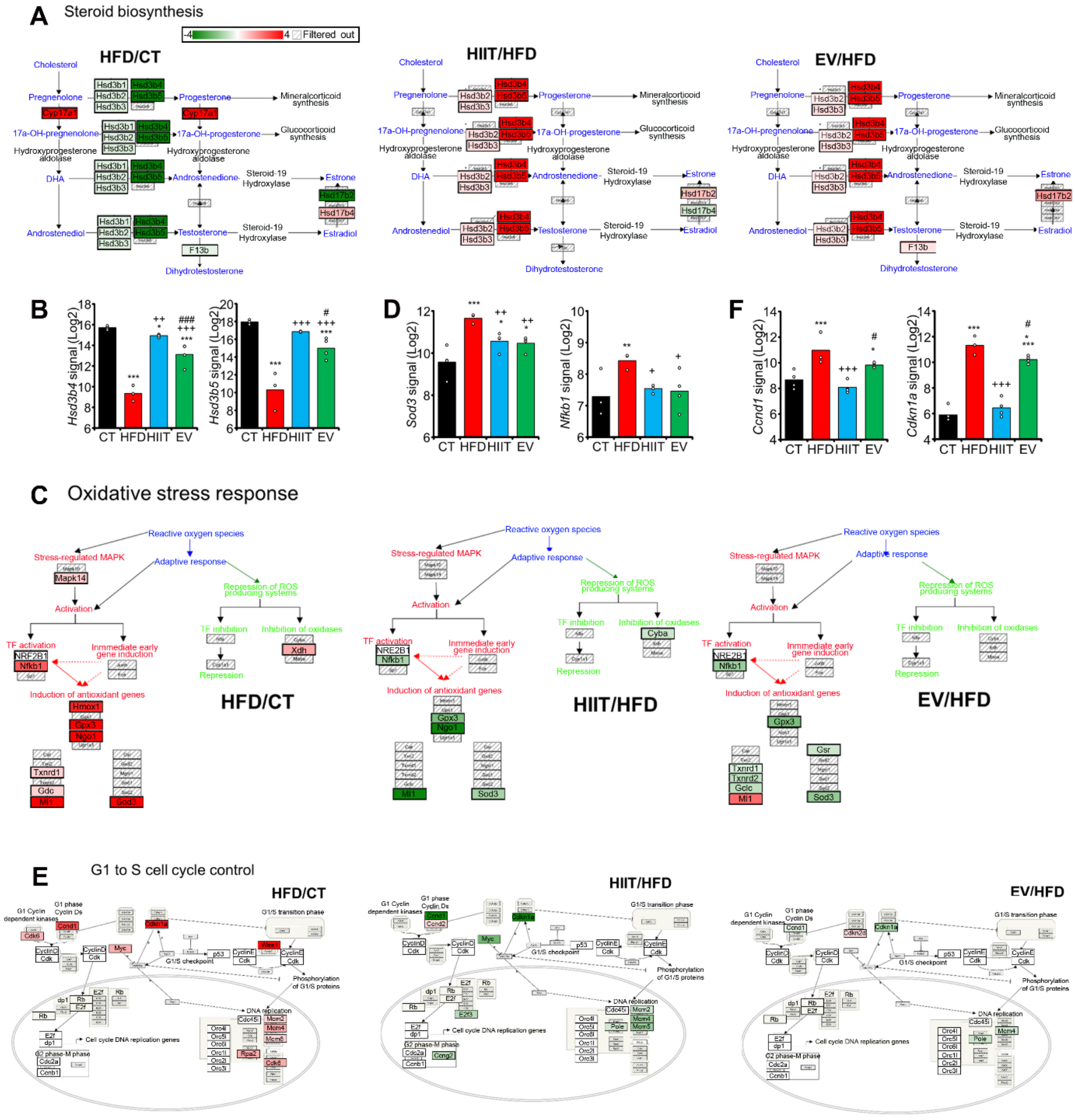

Supplementary Figure S7 continued in next page.

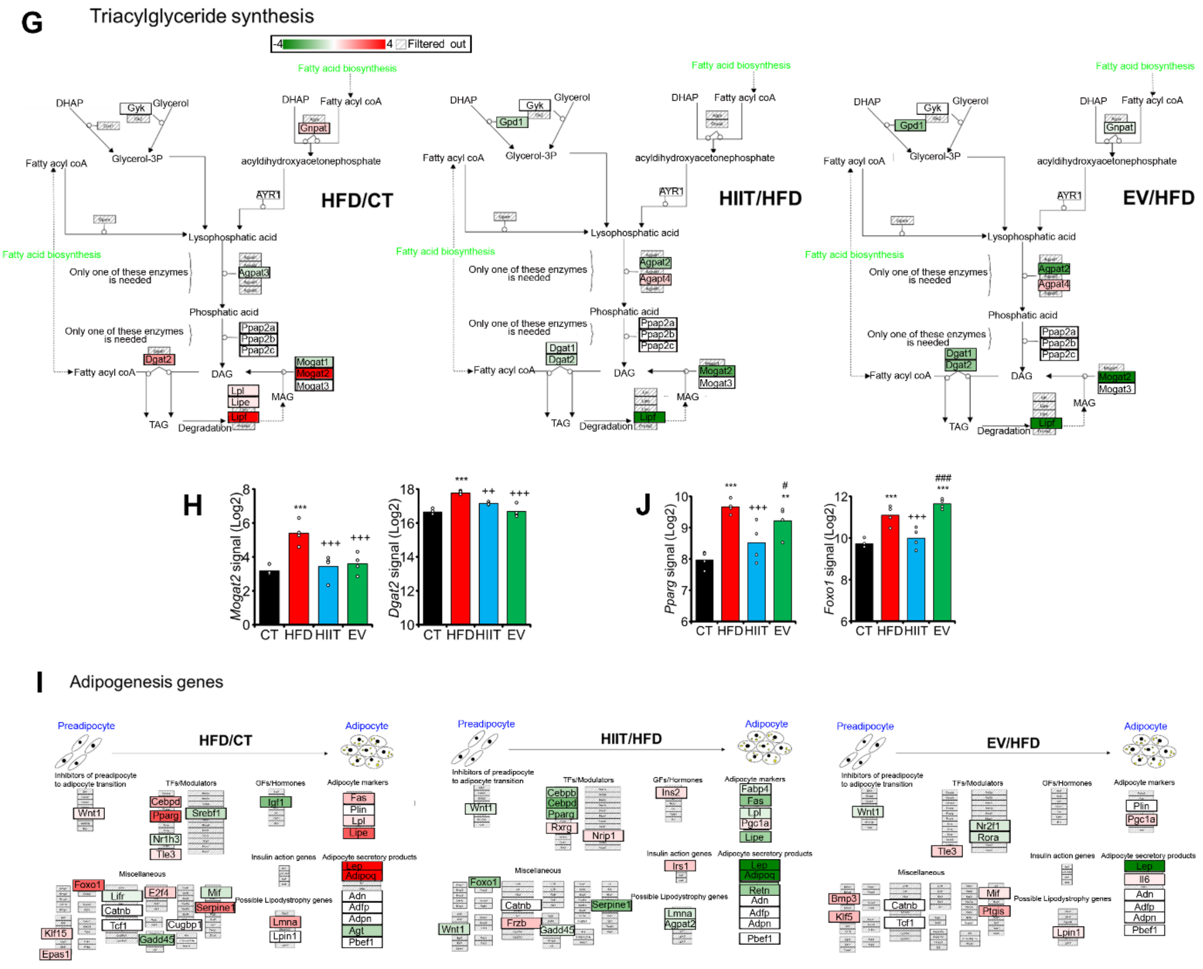

Supplementary Figure S7. EV-miRNAs regulate hepatic lipid metabolism to decrease steatosis. (A-F) Differentially enriched pathways with respect to HFD group and sample signals of the principal genes of interest in liver. The obesity-downregulated steroid biosynthesis pathway was increased by both HIIT and EV treatments **(A, B)**, that also decreased the obesity-upregulated oxidative stress response pathway **(C, D)**; on the other hand, the upregulated G1 to S cell cycle control pathway was normalized only by HIIT **(E, F)**. **(G, H)** The obesity-upregulated triacylglyceride synthesis pathway was reversed in sWAT by both HIIT and EV treatments. **(I, J)** The obesity-upregulated adipogenesis genes pathway in muscle was normalized only by HIIT. n=4/group (A-J). $^{**}p<0.01$, $^{***}p<0.005$ with respect to CT group. $^{+}p<0.05$, $^{++}p<0.01$, $^{+++}p<0.005$ with respect to HFD group. $^{\#}p<0.05$, $^{\#\#\#}p<0.005$ between HIIT and EV groups.
